# One-Pot Synthesis of Thiochromen-4-ones from 3-(Arylthio)propanoic Acids

**DOI:** 10.3390/chemistry7050163

**Published:** 2025-10-06

**Authors:** Kahlia S. Simpkins, Maggie Y. Guo, Toniyah D. Smith, Holden A. Hankerson, Fenghai Guo

**Affiliations:** 1 Department of Chemistry, Winston-Salem State University, 601 S. Martin Luther King Jr. Dr., Winston-Salem, NC 27110, USA; 2 Department of Chemistry, Wake Forest University, 1834 Wake Forest Road, Winston-Salem, NC 27109, USA

**Keywords:** thiochromen-4-ones, 3-(arylthio)propanoic acids, thiochroman-4-one, one-pot synthesis

## Abstract

Thiochromen-4-ones are known to possess useful optical properties and rich bioactivities, including antioxidant, antimicrobial, and anticancer properties. They are known to inhibit tumor cell growth, induce apoptosis, and have antiplatelet aggregation effects. Thiochromen-4-ones are also used as synthons and precursors in organic synthesis for bioactive agents. Although many synthetic approaches to oxygen-containing counterparts, chromones, have been reported, research on the synthesis of thiochromen-4-ones is scarce. The synthesis of thiochromen-4-ones can be challenging due to the inherent nature of sulfur, including its multiple oxidation states and tendency to form diverse bonding patterns. Here, we report the one-pot synthesis of thiochromen-4-ones, where two transformations of the starting material, 3-(arylthio)propanoic acid, are performed within a single reaction vessel, eliminating the need for an intermediate purification step. This one-pot reaction worked well with a variety of substrates with both electron-withdrawing and donating groups on the aromatic ring of 3-(arylthio)propanoic acids to give thiochromen-4-ones with good yields (up to 81%). This approach offers advantages like time and cost savings, increased efficiency, and reduced waste. This synthetic approach will allow access to a broader scope of thiochromen-4-ones due to the readily available thiophenols.

## 1. Introduction

**Figure 1.**
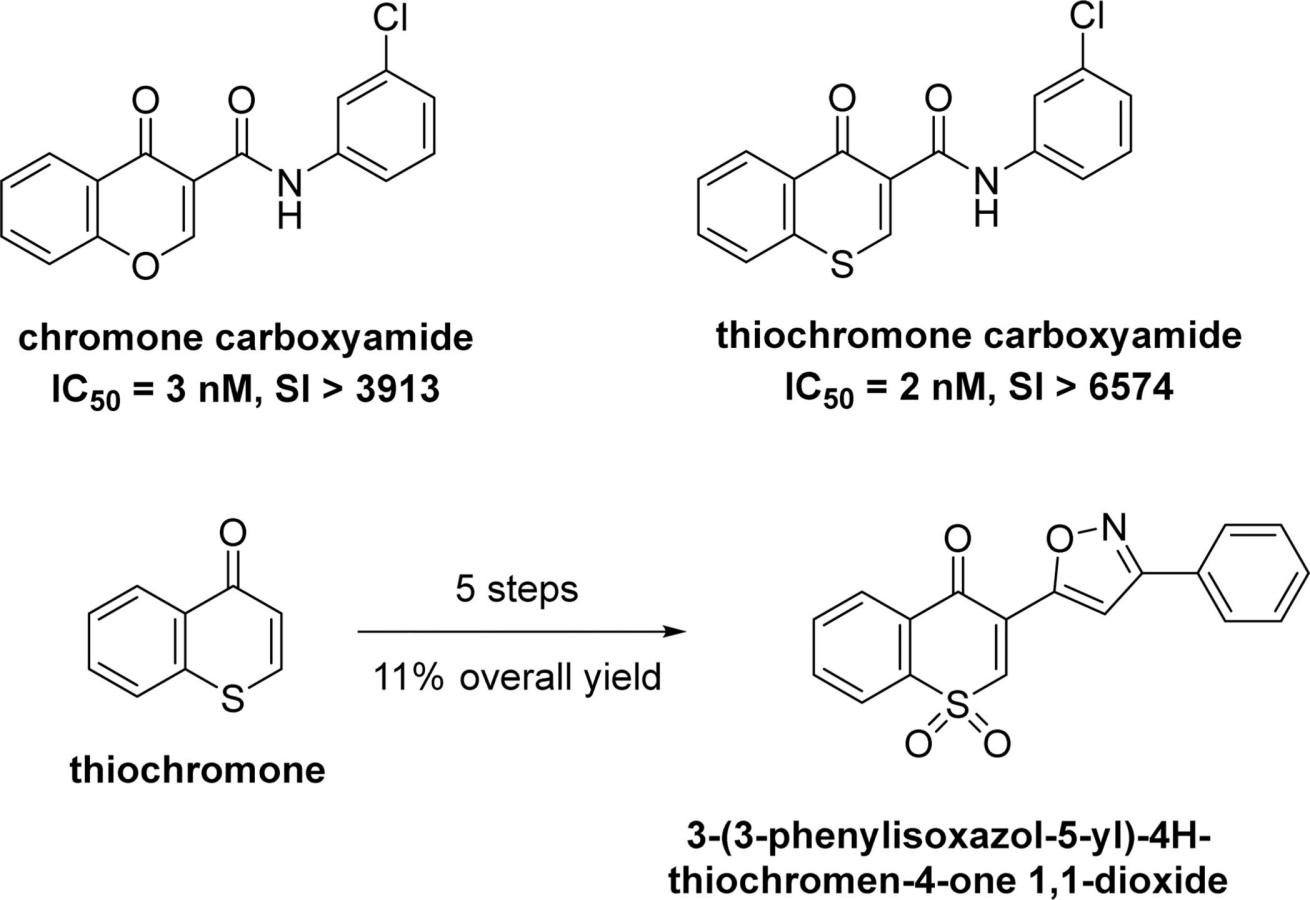
Chromone and thiochromone used as scaffolds as inhibitors for monoamine oxidase B.

Chromones are an important class of heterocycles and known as privileged scaffolds [1–3] in medicinal chemistry due to their wide range of biological activities including antioxidants [4], antiprotozoal [5], or anticancer agents [6–8]. Synthetic approaches to chromones have been extensively reported [9]. Thiochromen-4-ones [10] are the sulfur analogs of chromone, in which the O-1 atom is replaced by a sulfur atom. The sulfur-containing heterocyclic compounds, benzothiopyrans or thiochromen-4-ones stand out as having promising biological activities due to their structural relationship with chromones (benzopyrans), which are widely known as privileged scaffolds in medicinal chemistry [1–8]. However, the sulfur-containing thiochromen-4-ones have been significantly less explored, presumably due to the reduced reactivity of the thiopyrone moiety as the result of the replacement of oxygen by a sulfur atom. Thiochromen-4-ones are known to possess useful optical properties and rich bioactivities, including antioxidant, antimicrobial, and anticancer properties. They are known to inhibit tumor cell growth, induce apoptosis, and have antiplatelet aggregation effects [11,12]. Thiochromen-4-ones are also used as synthons and precursors in organic synthesis for useful sulfur heterocycles and other bioactive agents [13]. Chromone and thiochromen-4-ones are privileged scaffolds in medicinal chemistry. For example, chromone carboxamide and thiochromone carboxamide have been found to be selective inhibitors for human monoamine oxidases (hMAOs) [14]. A series of C-3 isoxazole-substituted thiochromone S,S-dioxide derivatives that exhibit strong inhibitory activity against hMAO-B have been synthesized using thiochrome as the key intermediate. 3-(3-Phenylisoxazol-5-yl)-4H-thiochromone 1,1-dioxide was synthesized in five steps using thiochromone as the key intermediate, with a good yield (Figure 1, 11% for five steps). In the past several years, we have reported the conjugate addition of organometallic reagents to thiochromen-4-ones in the synthesis of thioflavanones, thiochroman-4-ones with additional synthetic applications [15–18]. For example, thiochromen-4-ones have been reported to be used as the vital precursor for the synthesis of a variety of thioflavanones [18], an important class of sulfur-containing heterocycles with rich biological activities including the ability to significantly inhibit cellular proliferation with weak cytotoxicity and induce apoptosis in human breast cancer cells [19,20]. Thiochromen-4-one derivatives have also been synthesized and evaluated as potential Leishmanicidal agents [21].

**Figure 2.**
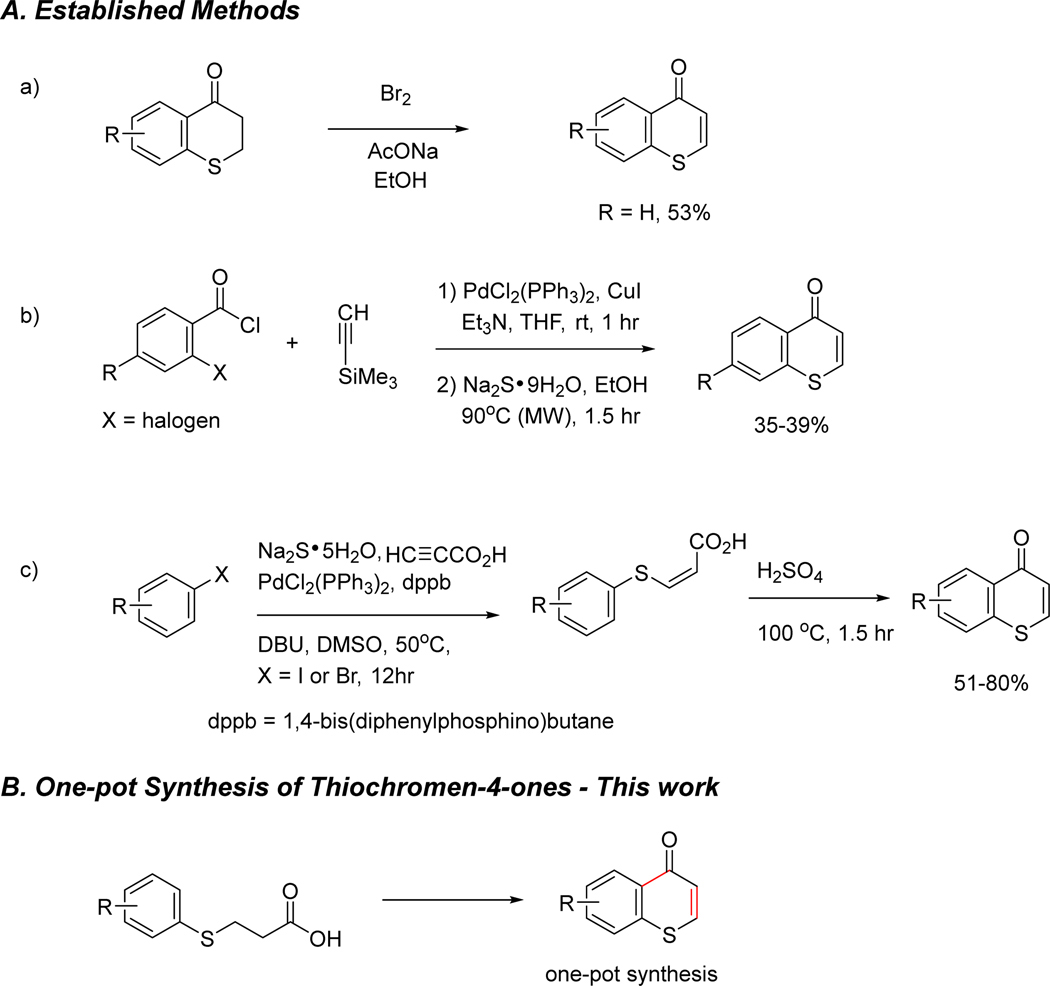
Synthesis of thiochromen-4-ones.

Although many synthetic approaches to oxygen-containing counterparts, chromones, have been reported [9], research on the synthesis of thiochromen-4-ones is scarce. The synthesis of thiochromen-4-ones can be challenging due to the inherent nature of sulfur, including its multiple oxidation states and tendency to form diverse bonding patterns. One of the earliest methods for the preparation of 2,3-unsubstituted thiochromone involved the bromination of thiochroman-4-one and subsequent dehydrohalogenation to give the desired thiochromone (Figure 2(Aa)) [22]. Another method utilized a 3-component synthesis of 2,3-unsubstituted thiochromen-4-ones from o-haloaroyl chlorides, trimethylsilylacetylene, and sodium sulfide nonahydrate with modest yields (35–39%, Figure 2(Ab)) [23,24]. Another synthetic approach to thiochromen-4-one involved a key intermediate, (*Z*)-3-arylthioacrylic acids, which were synthesized from aryl halides, sodium sulfide pentahydrate, and propiolic acid. The subsequent Friedel–Crafts acylation reaction of (*Z*)-3-arylthioacrylic acids under treatment with sulfuric acid at 100 °C produced thiochromen-4-ones in good yields (51–80%, Figure 2(Ac)) [25]. However, accessing a broad scope of thiochromen-4-ones, a class of sulfur-containing heterocycles, remains a significant synthetic challenge. Here, we report the one-pot synthesis [26] of thiochromen-4-ones (Figure 2B), where two transformations of the starting material, 3-(arylthio)propanoic acid, are performed within a single reaction vessel, eliminating the need for an intermediate purification step. This approach offers advantages like time and cost savings, increased efficiency, and reduced waste.

## 2. Materials and Methods

### 2.1. General Methods

The ^1^H and ^13^C-NMR spectra were recorded using a BRUKER Ascend^™^ 400 NMR spectrometer (Bruker corporation, Billerica, MA, USA), operating at 400 MHz for ^1^H and 100 MHz for ^13^C. Samples for NMR spectra were dissolved in deuterated chloroform (with TMS). Analytical thin layer chromatography (TLC) was performed on silica gel plates silica gel 60 F254 indicator. Visualization was accomplished with UV light (254 nm) and/or a 10% ethanol solution of phosphomolybdic acid and/or KMnO_4_ stain prepared by dissolving 1.5 g KMnO_4_, 10 g potassium carbonate, and 1.25 mL 10% sodium hydroxide in 200 mL water. Flash chromatography was performed with 200–400 mesh silica gel.

### 2.2. Materials

Chemicals and solvents were obtained from commercial sources and used without further purification unless stated otherwise.

### 2.3. *Preparation of Starting 3-(Arylthio)propanoic Acids* 1

**Scheme 1.**
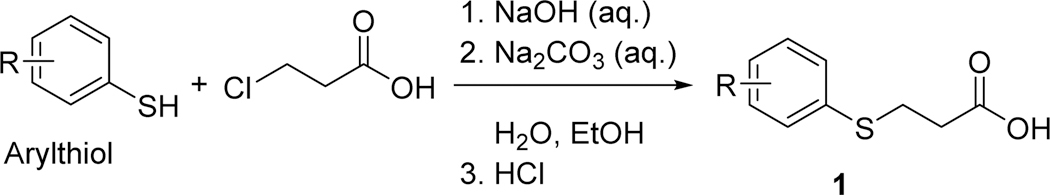
Preparation of 3-(arylthio)propanoic acid.

The starting 3-(arylthio)propanoic acids **1** were prepared according to the procedure previously reported in the literature with good chemical yields (Scheme 1, 80–93%) [27,28]. Arylthiols were purchased from a commercial source and used as received without further purification. A 250 mL flask was charged with a stirrer bar, with aqueous NaOH (25 mL, 1.0 M) and aqueous Na_2_CO_3_ (25 mL, 1.0 M). To the above solution, arylthiols (50 mmol) was then added as a solution in 30 mL of EtOH followed by the addition of 3-chloropropanoic acid (51 mmol) as an aqueous solution in 20 mL of water. The resultant reaction mixture was stirred at room temperature for 2 h, then heated to reflux in an oil bath overnight (12 h). The resultant mixture was then cooled down to room temperature, EtOH was evaporated under a rotovap, then the aqueous phase was acidified to pH 1~2 with conc. HCl (6.0 M). The solution was diluted with H_2_O (30 mL) and extracted with CH_2_Cl_2_ three times (3 ×40 mL), and the organic layers were combined, dried (Na_2_SO_4_), filtered, and concentrated to obtain the crude product. This was then purified using flash column chromatography (ethyl acetate/hexanes, 5% to 30%) to obtain the desired 3-(arylthio)propanoic acid **1** with a good chemical yield (Scheme 1, 80–93%).

### 2.4. General Procedures

#### General Procedure A

A round-bottom flask with a stir bar was charged with 3-(arylthio)propanoic acids (**1**, 1.0 mmol, 1.0 equivalent, Scheme 1), then dichloromethane (1.0 mL) and polyphosphoric acid (PPA, 0.5 mL, excess) were added. DCM was added to dissolve the solid starting material, which was initially mixed well with viscous PPA at room temperature. The DCM was then distilled and collected in a collecting flask when the reaction mixture was heated to the boiling point of DCM (40 °C). The resulting mixture was then heated to 100 °C (oil bath temperature), and the reaction was monitored using TLC. Once the TLC monitoring showed complete consumption of the starting material, the reaction mixture was allowed to cool down to room temperature. An aqueous saturated NaHCO_3_ solution was then added dropwise (5.0 mL), and the resultant mixture was allowed to stir for 2 h at room temperature. It was then extracted with dichloromethane (3 × 15.0 mL). The organic layers were combined, dried with Na_2_SO_4_, filtered, and evaporated by vacuum to give a crude product. The crude product was purified using column chromatography with a silica gel with a mixture of hexanes/ethyl acetate as the eluent to give the product thiochromen-4-ones **3** (Scheme 1) with a 55–81% yield.

### 2.5. Synthesis

HRMS data for compounds **3d**, **3f**, **3g**, **3m** were analyzed using TOF MS. Compounds **3a**, **3b**, **3c**, **3e**, and **3h**–**l** have been fully characterized and reported [25]. (The NMR spectra and Mass spectra for new compounds are included in the Supplementary Materials. The copies of NMR spectra compounds **3a**, **3b**, **3c**, **3e**, and **3h**–**l** are also included in the Supplementary Materials to verify the purity and structure).

#### 2.5.1. Synthesis of 6-Methoxyl-4H-Thiochromen-4-one (3a)

Employing General Procedure A and using 3-(4-methoxylphenyl)propanoic acids (212 mg, 1.0 mol), purification via flash column chromatography (silica, 5–10% Ethyl acetate: hexanes, *v*/*v*) produced grey solid **3a** (155 mg, 81%): mp 110–111 °C. ^1^H NMR (400 MHz, CDCl_3_): δ 3.92 (s, 3H), 7.13 (dd, *J* = 0.8, 10.4 Hz, 1 H), 7.26 (dd, *J* = 2.8, 8.8 Hz, 1 H), 7.55 (d, *J* = 8.8 Hz, 1 H), 7.91 (d, *J* = 10.4 Hz, 1 H), 7.99 (d, *J* = 2.8 Hz, 1 H); ^13^C NMR (100 MHz, CDCl_3_): δ 56.1, 108.7, 123.5, 123.7, 128.3, 131.3, 132.4, 141.3, 160.4, 179.0; HRMS (EI-ion trap) *m*/*z*: [M]^+^ calcd. for C_10_H_8_O_2_S, 192.0245; found 192.0241.

#### 2.5.2. Synthesis of 8-Methylthiochromen-4-one (3d)

Employing General Procedure A and using 3-(2-methylphenylthio)propanoic acids (196 mg, 1.0 mol), purification using flash column chromatography (silica, 5–10% Ethyl acetate: hexanes, *v*/*v*) produced white solid **3d** (119 mg, 68%): mp 96.5–97.0 °C. ^1^H NMR (400 MHz, CDCl_3_): δ 2.30 (s, 3H), 6.85 (d, *J* = 10.4 Hz, 1 H), 7.19–7.28 (m, 2 H), 7.64 (d, *J* = 10.4 Hz, 1 H), 8.21 (ddd, *J* = 0.8, 2.4, 7.2 Hz, 1 H); ^13^C NMR (100 MHz, CDCl_3_): δ 19.7, 125.6, 126.7, 127.4, 132.6, 132.8, 135.0, 137.4, 137.8, 180.3; HRMS (EI-ion trap) *m*/*z*: [M]^+^ calcd. for C_10_H_8_OS, 176.0296; found 176.0293.

#### 2.5.3. Synthesis of 6,8-Dimethylthiochromen-4-one (3f)

Employing General Procedure A and using 3-(2,4-dimethylphenylthio)propanoic acids (210 mg, 1.0 mol), purification using flash column chromatography (silica, 5–10% Ethyl acetate: hexanes, *v*/*v*) produced light yellow solid **3f** (133 mg, 70%): mp 102–103 °C. ^1^H NMR (400 MHz, CDCl_3_): δ 2.38 (s, 3H), 2.42 (s, 3H), 6.97 (d, *J* = 10.4 Hz, 1 H), 7.24 (s, 1 H), 7.78 (d, *J* = 10.4 Hz, 1 H), 8.18 (s, 1 H); ^13^C NMR (100 MHz, CDCl_3_): δ 19.6, 21.3, 125.5, 126.4, 132.6, 134.2, 134.4, 134.8, 137.3, 137.5, 180.4; HRMS (EI-ion trap) *m*/*z*: [M]^+^ calcd. for C_11_H_10_OS, 190.0452; found 190.0455.

#### 2.5.4. Synthesis of 8-Isopropylthiochromen-4-one (3g)

Employing General Procedure A and using 3-(2-methylphenylthio)propanoic acids (224 mg, 1.0 mol), purification using flash column chromatography (silica, 5–10% Ethyl acetate: hexanes, *v*/*v*) produced light yellow solid **3g** (126 mg, 62%): mp 83–84 °C. ^1^H NMR (400 MHz, CDCl_3_): δ 1.39 (d, *J* = 6.8 Hz, 6H), 3.43 (septet, *J* = 6.8 Hz, 1H), 7.04 (d, *J* = 10.4 Hz, 1 H), 7.55 (t, *J* = 8 Hz, 1 H), 7.61 (dd, *J* = 1.6, 7.2 Hz, 1 H), 7.88 (d, *J* = 10.4 Hz, 1 H), 8.48 (dd, *J* = 1.6, 8.0 Hz, 1 H); ^13^C NMR (100 MHz, CDCl_3_): δ 23.2, 30.5, 124.5, 127.0, 128.1, 128.7, 132.2, 136.7, 139.4, 145.8, 180.5; HRMS (EI-ion trap) *m*/*z*: [M]^+^ calcd. for C_12_H_12_OS, 204.0609; found 204.0611.

#### 2.5.5. Synthesis of 8-Methoxylthiochromen-4-one (3m)

Employing General Procedure A and using 3-(2-methoxylphenylthio)propanoic acids (212 mg, 1.0 mol), purification using flash column chromatography (silica, 5–10% Ethyl acetate: hexanes, *v*/*v*) produced grey solid **3m** (140 mg, 73%): mp 136–137 °C. ^1^H NMR (400 MHz, CDCl_3_): δ 4.00 (s, 3H), 7.06–7.18 (m, 2 H), 7.50 (t, *J* = 8.0 Hz, 1 H), 7.92 (d, *J* = 10.4 Hz, 1 H), 8.16 (dd, *J* = 0.8, 8.0 Hz, 1 H); ^13^C NMR (100 MHz, CDCl_3_): δ 56.7, 111.7, 120.7, 125.2, 128.2, 128.7, 133.0, 139.6, 155.1, 179.8; HRMS (EI-ion trap) *m*/*z*: [M]^+^ calcd. for C_10_H_8_O_2_S, 192.0245; found 192.0249.

#### 2.5.6. Synthesis of 4H-Thiochromen-4-one (3b)

Employing General Procedure A and using 3-(phenyl)propanoic acids (182 mg, 1.0 mol), purification using flash column chromatography (silica, 5–10% Ethyl acetate: hexanes, *v*/*v*) produced **3b** as light yellow solid (111 mg, 69%), mp 93–94 °C. ^1^H NMR (400 MHz, CDCl_3_): δ 7.07 (d, *J* = 10.40 Hz, 1H), 7.51–7.62 (m, 3H), 7.61 (dd, *J* = 1.20, 4.80 Hz, 1H), 7.85 (d, *J* = 10.40 Hz, 1H), 8.53 (m, 1 H); ^13^C NMR (100 MHz, CDCl3): δ 125.8, 126.9, 127.7, 128.2, 131.7, 132.2, 137.9, 138.6, 179.9; HRMS (EI-ion trap) *m*/*z*: [M] + calcd. for C_9_H_6_OS, 162.0139; found 162.0132.

#### 2.5.7. Synthesis of 6-Methylthiochromen-4-one (3c)

Employing General Procedure A and using 3-(4-methylphenyl) propanoic acids (196 mg, 1.0 mol), purification using flash column chromatography (silica, 5–10% Ethyl acetate: hexanes, *v*/*v*) produced a light yellow solid **3c** (126 mg, 72%), mp 69–70 °C. ^1^H NMR (400 MHz, CDCl_3_): δ 2.46 (s, 3 H), 7.06 (d, *J* = 10.40 Hz, 1 H), 7.42 (d, *J* = 2.0, 8.0 Hz, 1 H), 7.50 (d, *J* = 8.40 Hz, 1 H), 7.84 (d, *J* = 10.40 Hz, 1 H), 8.34 (dd, *J* = 0.8, 1.20 Hz, 1 H); ^13^C NMR (100 MHz, CDCl_3_): δ 21.5, 125.5, 126.7, 128.5, 131.9, 133.2, 134.9, 138.6, 138.7, 179.8.

#### 2.5.8. Synthesis of 6,7-Dimethylthiochromen-4-one (3e)

Employing General Procedure A and using 3-(3,4-dimethylphenyl)propanoic acids (210 mg, 1.0 mol), purification using flash column chromatography (silica, 5–10% Ethyl acetate: hexanes, *v*/*v*) produced a light brown solid **3e** (119 mg, 63%), mp 143–144 °C. IR (neat) 3008 (s), 2916 (s), 1617 (s), 1589 (s), 1481 (m), 1400 (m), 1376 (m), 1145 (m), 1105 (m), 1023 (w), 826 (m) cm^−1^; ^1^H NMR (400 MHz, CDCl_3_): δ 2.38 (s, 3H), 2.39 (s, 3 H), 7.16 (d, *J* = 6.40 Hz, 1 H), 7.41 (s, 1H), 7.88 (d, *J* = 10.40 Hz, 1 H), 8.31 (s, 1H); ^13^C NMR (100 MHz, CDCl_3_): δ 19.6, 20.0, 124.5, 126.8, 128.6, 129.2, 135.7, 138.2, 139.5, 142.6, 179.3; HRMS (EI-ion trap) *m*/*z*: [M]^+^ calcd. for C_11_H_10_OS, 190.0452; found 190.0449.

#### 2.5.9. Synthesis of 6-*tert*-Butylthiochromen-4-one (3h)

Employing General Procedure A and using 3-(4-t-butylphenyl)propanoic acids (238 mg, 1.0 mol), purification using flash column chromatography (silica, 5–10% Ethyl acetate: hexanes, *v*/*v*) produced a light yellow oil **3g** (141 mg, 65%). 1H NMR (400 MHz, CDCl_3_): δ 1.37 (s, 9H), 7.09 (dd, *J* = 2.4, 10.40 Hz,1H), 7.56 (d, *J* = 8.40 Hz, 1 H), 7.68 (dd, *J* = 2.40, 8.80 Hz, 1 H), 7.86 (d, *J* = 10.40 Hz, 1 H), 8.55 (d, *J* = 1.60 Hz, 1 H); ^13^C NMR (100 MHz, CDCl_3_): δ 31.1, 35.1, 124.7, 125.3, 126.5, 129.7, 131.5, 134.9 137.5, 151.7, 179.9; HR-MS (ESI): *m*/*z* = 218.0765, calcd. for C_13_H_14_OS, found [M]^+^ 218.0767.

#### 2.5.10. Synthesis of 6-Fluorothiochromen-4-one (3i)

Employing General Procedure A and using 3-(4-fluoro)propanoic acids (200 mg, 1.0 mol), purification using flash column chromatography (silica, 5–10% Ethyl acetate: hexanes, *v*/*v*) produced a grey solid **3i** (99 mg, 55%), mp 155–156 °C. ^1^H NMR (300 MHz, CDCl_3_): δ 7.00 (d, *J* = 10.40 Hz, 1H), 7.33–7.40 (m, 1 H), 7.61 (dd, *J* = 4.80, 8.80 Hz, 1H), 7.85 (d, *J* = 10.40 Hz, 1 H), 8.21 (dd, *J* = 2.8 Hz, 9.60 Hz, 1 H); ^13^C NMR (75 MHz, CDCl_3_): δ 114.4 (d, *J_C,F_* = 23 Hz), 120.6 (d, *J_C,F_* = 24 Hz), 125.1, 129.1 (d, *J_C,F_* = 8 Hz), 133.0, 134.5, 138.1, 162.3 (d, *J_C,F_* = 248 Hz), 179.0 (d, *J_C,F_* = 3.0 Hz), HR-MS (ESI): *m*/*z* = 180.0045, calcd. for C_9_H_5_OFS, found [M]^+^ 180.0040.

#### 2.5.11. Synthesis of 6-Bromothiochromen-4-one (3j)

Employing General Procedure A and using 3-(4-bromo)propanoic acids (260 mg, 1.0 mol), purification using flash column chromatography (silica, 5–10% Ethyl acetate: hexanes, *v*/*v*) produced a crimson solid **3i** (139 mg, 58%), mp 156–157 °C. ^1^H NMR (400 MHz, CDCl_3_): δ 7.02 (d, *J* = 10.40 Hz, 1H), 7.45 (d, *J* = 8.40 Hz, 1 H), 7.70 (dd, *J* = 2.00, 8.80 Hz, 1 H), 7.82 (d, *J* = 10.40 Hz, 1 H), 8.67 (d, *J* = 2.40 Hz, 1H); ^13^C NMR (100 MHz, CDCl_3_): δ 122.4, 126.0, 128.4, 131.6, 133.7, 134.8, 136.3 138.0, 178.6; HR-MS (ESI): *m*/*z* = 239.9244, calcd. for C_9_H_6_BrOS, found [M]^+^: 239.9240.

#### 2.5.12. Synthesis of 6-Chlorothiochromen-4-one (3k)

Employing General Procedure A and using 3-(4-chloro)propanoic acids (214 mg, 1.0 mol), purification using flash column chromatography (silica, 5–10% Ethyl acetate: hexanes, *v*/*v*) produced a white solid **3i** (117 mg, 60%), mp 146–147 °C. ^1^H NMR (400 MHz, CDCl_3_): δ 7.01 (d, *J* = 10.40 Hz, 1H), 7.56–7.59 (m, 2 H), 7.82 (d, *J* = 10.40 Hz, 1 H), 8.52 (dd, *J* = 1.2, 2.00 Hz, 1 H); ^13^C NMR (100 MHz, CDCl_3_): δ 125.9 128.3, 128.5, 132.1, 133.6, 134.6, 135.8, 138.0, 178.7; HR-MS (ESI): *m*/*z* = 195.9750, calcd. for C_9_H_5_ClOS, found [M]^+^: 195.9746.

#### 2.5.13. Synthesis of 6-(Trifluoromethyl)thiochromen-4-one (3l)

Employing General Procedure A and using 3-(4-trifluoromethyl)propanoic acids (250 mg, 1.0 mol), purification using flash column chromatography (silica, 5–10% Ethyl acetate: hexanes, *v*/*v*) produced a crimson solid **3i** (128 mg, 56%), mp 79–80 °C. ^1^H NMR (300 MHz, CDCl_3_): δ 7.04 (d, *J* = 10.4 Hz, 1H), 7.72 (d, *J* = 8.4 Hz, 1H), 7.79 (dd, *J* = 2.0, 8.80 Hz, 1H), 7.83 (d, *J* = 10.4 Hz, 1H), 8.80 (dd, *J* = 0.8, 1.2 Hz, 1H); ^13^C NMR (75 MHz, CDCl_3_): δ 123.6 (q, *J_C,F_* = 271 Hz), 126.4 (q, *J_C,F_* = 4.0 Hz), 126.5, 127.6 (q, *J_C,F_* = 3.0 Hz), 127.8, 130.3 (q, *J_C,F_* = 34.0 Hz), 132.5, 138.0, 141.2, 178.8; HR-MS (ESI): *m*/*z* = 230.0013, calcd. for C_10_H_5_F_3_OS, found [M]^+^: 230.0008.

## 3. Results and Discussion

The starting 3-(arylthio)propanoic acids **1** were prepared according to the procedure previously reported in the literature, with good yields (Scheme 1, 80–93%) [27,28].

We started our investigation with 3-(4-methoxyphenylthio)propanoic acid **1a** and concentrated sulfuric acid. Upon treatment of 3-(4-methoxyphenylthio)propanoic acid **1a** in DCM (1.0 mL) with excess concentrated sulfuric acid (95–97%, 0.5 mL) at 0 °C in an ice bath, no desired thiochromen-4-one **3a** was observed after warming up to room temperature and stirring for 12 h. Instead, it was found that the demethylated byproduct thiochroman-4-one **2b** was attained with a modest yield (Table 1, entry 1, 45%). Then, an excess amount of concentrated phosphoric acid (85%, 0.5 mL) was added to 3-(4-methoxyphenylthio)propanoic acid **1a** in DCM (1.0 mL). A similar result was attained, as no desired thiochromen-4-one **3a** was observed, and only demethylated thiochroman-4-one **2b** was attained (Table 1, entry 2, 40%). These results indicate that strong acids such as concentrated sulfuric acid and phosphoric acid are not tolerant with methoxy (MeO-) substituent on the 3-(4-methoxyphenylthio)propanoic acid **1a**. Therefore, a weaker acid polyphosphoric acid (PPA) was then deployed instead. When an excess amount of polyphosphoric acid (PPA, 0.5 mL) was added under similar conditions (DCM as solvent, 0 °C using an ice bath), no reaction was observed after warming to room temperature and stirring for 12 h, as we only recovered the starting material (Table 1, entry 3). When the reaction mixture was heated to the boiling point of DCM (40 °C) (Table 1, entries 4–8), the solvent DCM was distilled and collected in a collecting flask. Subsequently, when the reaction was heated to 50 °C for 2 h with excess polyphosphoric acid (PPA, 0.5 mL), the corresponding thiochroman-4-one **2a** was attained with a low yield (entry 4, 20%), but no desired thiochromen-4-one **3a** was observed. A slightly higher yield of thiochromone-4-one **2a** was attained when the reaction was heated to 50 °C for 4 h with excess polyphosphoric acid (PPA, 0.5 mL) (entry 5, 25%, no desired thiochromen-4-one **3a**). Although no desired thiochromen-4-one **3a** was observed, the yield of thiochromone-4-one **2a** was significantly increased when the reaction mixture was heated to 50 °C for an extended period of time (entry 6, 12 h). Finally, we were delighted to see the formation of thiochromen-4-one **3a** when the reaction temperature was further increased to 100 °C (entry 7). The highest yield was attained with 3-(4-methoxyphenylthio)propanoic acid and polyphosphoric acid (PPA) at 100 °C for 12 h (entry 8). It was found that polyphosphoric acid (PPA) is required for the one-pot reaction to happen (entry 9, no PPA acid, no thiochromen-4-one, 0%). Interestingly, this one-pot reaction also worked under argon to produce a comparable yield of thiochromen-4-one **3a** (etntry 10, 79%). It was found that the reaction also worked well without DCM (entry 11, 75%).

**Scheme 2.**
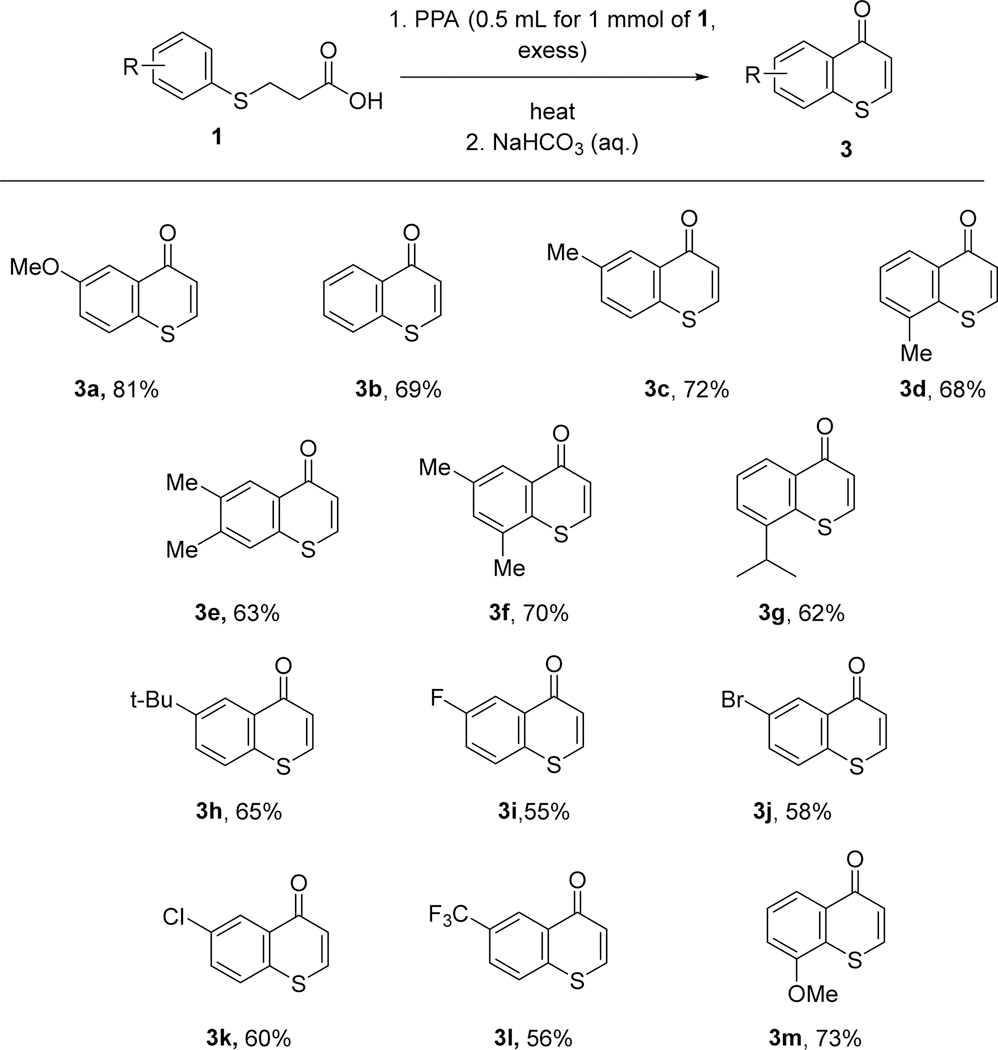
The scope of the one-pot reaction of 3-(arylthio)propanoic acids.

Having found the optimal reaction condition for the one-pot synthesis of thiochromen-4-one, we next turned our attention to explore the scope of this reaction. A variety of 3-(arylthio)propanoic acids with both electron-withdrawing and donating group were investigated. It was found that 3-(arylthio)propanoic acids with both electron-withdrawing and donating groups on the aromatic ring underwent the one-pot reaction to afford thiochromen-4-ones **3a**–**3m** with 55–81% yields (Scheme 2). 3-(Phenylthio)propanoic acid worked well under the optimal one-pot synthesis reaction condition to give thiochromen-4-one **3b** with a good yield. 3-(Arylthio)propanoic acids bearing simple alkyl substituents on the aromatic ring, such as methyl groups, reacted well to afford **3c**–**f** in 63–72% yields (Scheme 2). Steric hindrance was not a factor, as slightly bulky substituents such as *i*-Pr worked well to furnish the desired thiochromen-4-one **3g** with a 62% yield. The bulky *t*-butyl group was also tolerated to afford the corresponding **3h** with a good yield (Scheme 2, 65%). 3-(Arylthio)propanoic acids with halides F, Br, and Cl also worked well under this one-pot reaction condition, but with slightly lower chemical yields (Scheme 2, 55–60%). The strong electron-withdrawing group trifluoromethyl also worked with a slightly lower chemical yield (Scheme 2, **3l**, 56%). 3-(Arylthio)propanoic acids with electron-donating groups, such as MeO-, also worked well to afford thiochromen-4-ones **3a** (81%) and **3m** (73%) in good yields (Scheme 2).

## 4. Conclusions

In conclusion, we successfully developed the one-pot synthesis of thiochromen-4-ones from 3-(arylthio)propanoic acids. This reaction was shown to work well with a broad range of substrates with both electron-withdrawing and donating groups on the aromatic ring of 3-(arylthio)propanoic acids. 3-(Arylthio)propanoic acids with both electron-withdrawing and donating groups on the aromatic ring underwent the one-pot reaction to afford thiochromen-4-ones with good chemical yields (55–81% in one-pot synthesis). With this one-pot approach, two transformations were performed within a single reaction vessel, eliminating the need for an intermediate purification step. This one-pot approach offers advantages like time and cost savings, increased efficiency, and reduced waste. This synthetic approach will allow access to a broader scope of thiochromen-4-ones due to the readily available thiophenols.

## Supplementary Material

Supplementary material

**Supplementary Materials:** The following supporting information can be downloaded at: https://www.mdpi.com/article/10.3390/chemistry7050163/s1.

## Figures and Tables

**Table 1.** Reactions with 3-(4-methoxyphenylthio)propanoic acid.

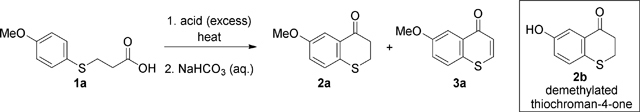						
entry	acid ^a^	T (°C)	Time (hr)	2a yield (%) ^b^	2b (%)	3a (%) ^b^
1	H_2_SO_4_	0 to RT	12	0	45	0
2	H_3_PO_4_	0 to RT	12	0	40	0
3	PPA	0 to RT	12	0		0
4	PPA	50	2	20		0
5	PPA	50	4	25		0
6	PPA	50	12	83		0
7	PPA	100	5	35		30
8	PPA	100	12			81
9	-	100	12			0
10	PPA	100	12			79 ^c^
11	PPA	100	12			75 ^d^

a Excess amount of acid was used (0.5 mL acid for 1.0 mmol of starting material 1a).

b Yields are based on isolated products determined via column chromatography.

c The reaction was run under Argon.

d No DCM added.

## Data Availability

The data is available in the supporting information.
