# Supplementary material for: One-Pot Synthesis of Thiochromen-4-ones from 3-(Arylthio)propanoic Acids
Source: Chemistry (Basel). Author manuscript; Available in PMC 2025 Dec 13. (PMC12700290; doi:10.3390/chemistry7050163)
Supplement: Supplementary material [file NIHMS2116949-supplement-Supplementary_material.pdf]

One-pot Synthesis of Thiochromen-4-ones from 3-(arylthio)propanoic acids

Kahlia S. Simpkins,[a] Maggie Y. Guo,[a] Toniyah D. Smith,[a] Holden A. Hankerson,[b] and Fenghai Guo[a,*]

[a] Department of Chemistry, Winston-Salem State University, 601 S. Martin Luther King Jr. Dr., Winston-Salem, NC 27110, USA

[b] Department of Chemistry, Wake Forest University, 1834 Wake Forest Road, Winston-Salem, NC 27109

Supporting Information

^{1}H, and ^{13}C-NMR spectra **S2-S11**

^{1}H for **3a** S2

^{13}C for **3a** S3

^{1}H for **3d** S4

^{13}C for **3d** S5

^{1}H for **3f** S6

^{13}C for **3f** S7

^{1}H for **3g** S8

^{13}C for **3g** S9

^{1}H for **3m** S10

^{13}C for **3m** S11

Mass spectra S12-S16

Mass spectra: 3a – S12**, 3d –** S13**, 3f –** S14**, 3g –** S15**, 3m –** S16**.**

^{1}H, and ^{13}C-NMR spectra for compounds 3b-c, 3e, 3h-l reported in literature S17-S32

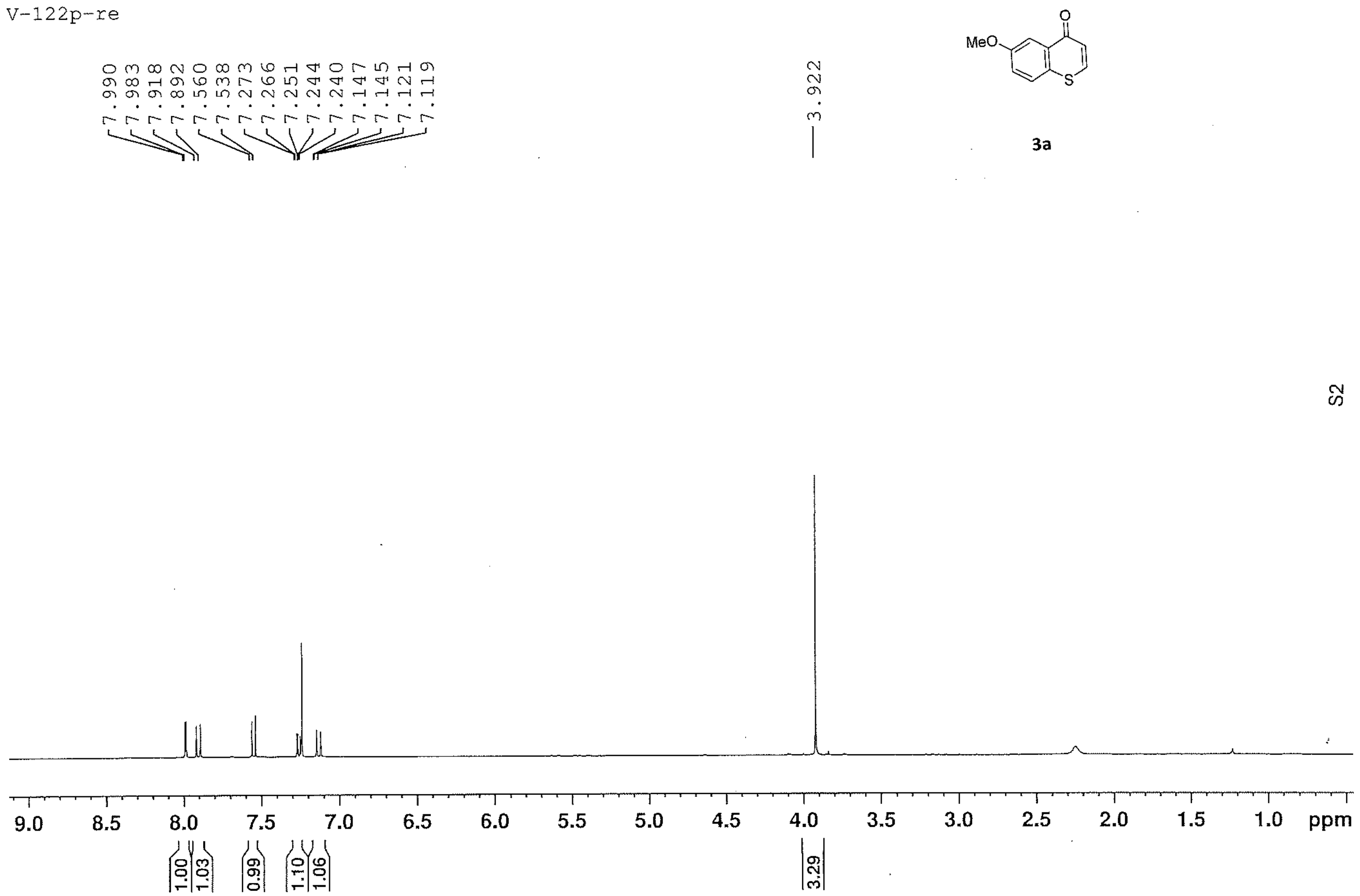
V-122p-re
7.990
7.983
7.918
7.892
7.560
7.538
7.273
7.266
7.251
7.244
7.240
7.147
7.145
7.121
7.119
3.922
MeO
S
O
3a
9.0
8.5
8.0
7.5
7.0
6.5
6.0
5.5
5.0
4.5
4.0
3.5
3.0
2.5
2.0
1.5
1.0
ppm
1.00
1.03
0.99
1.10
1.06
3.29

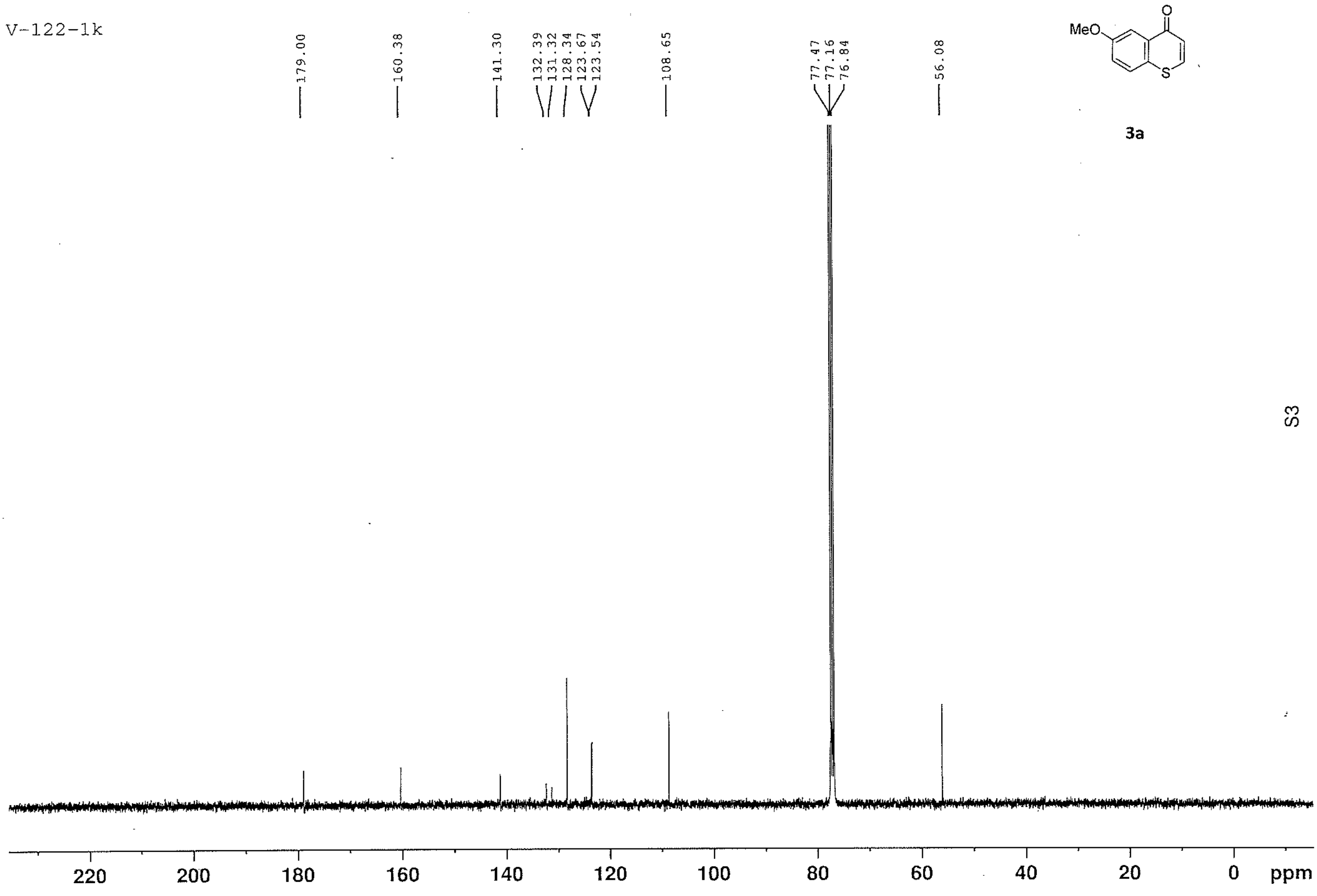
V-122-1k
179.00
160.38
141.30
132.39
131.32
128.34
123.67
123.54
108.65
77.47
77.16
76.84
56.08
MeO
O
S
220
200
180
160
140
120
100
80
60
40
20
0
ppm

3a

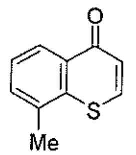

3d

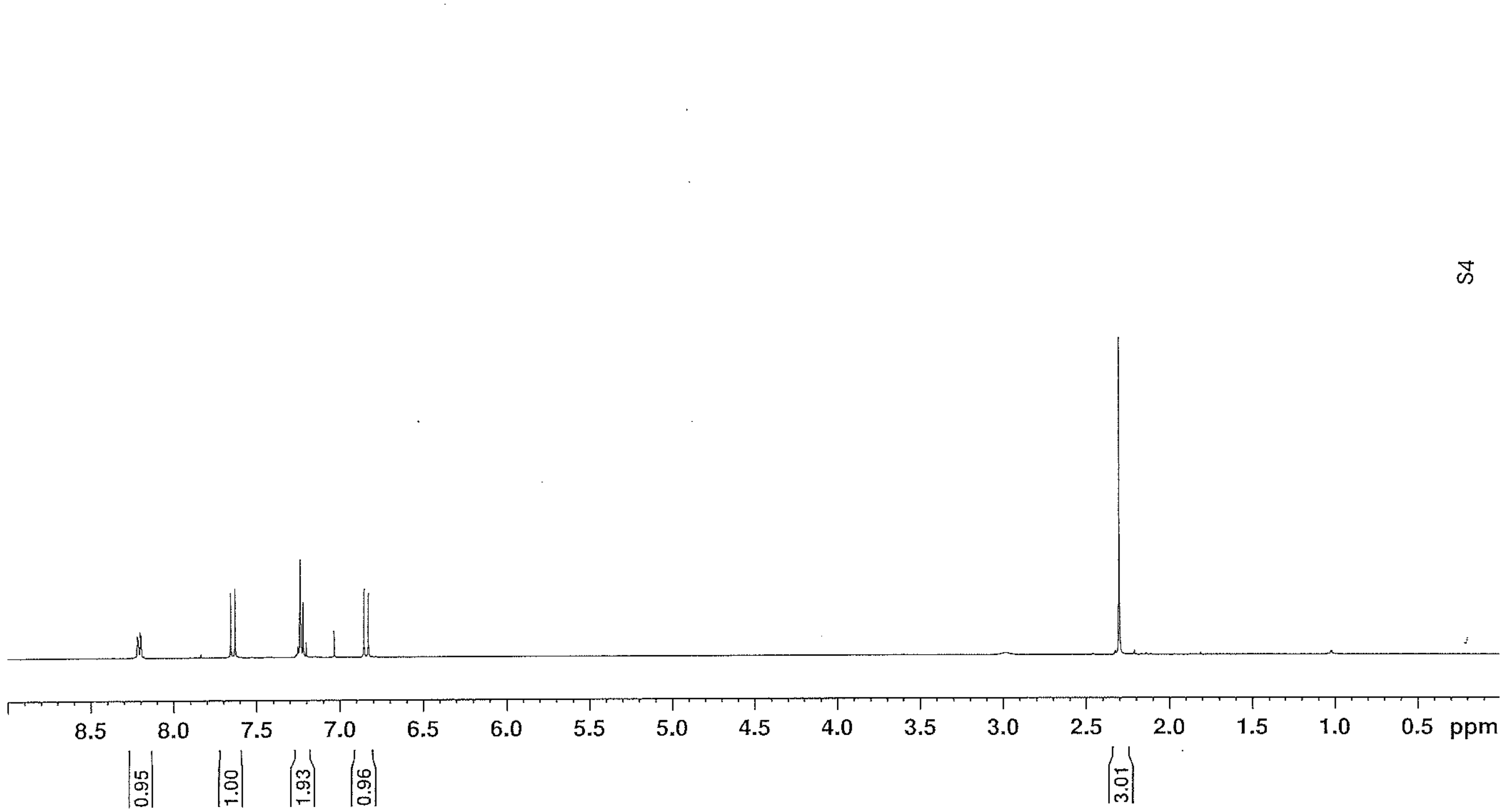

II-76rere
8.221
8.220
8.215
8.213
8.203
8.197
7.657
7.631
7.254
7.253
7.240
7.236
7.222
7.204
7.036
2.299
8.5
8.0
7.5
7.0
6.5
6.0
5.5
5.0
4.5
4.0
3.5
3.0
2.5
2.0
1.5
1.0
0.5
ppm
0.95
1.00
1.93
0.96
3.01

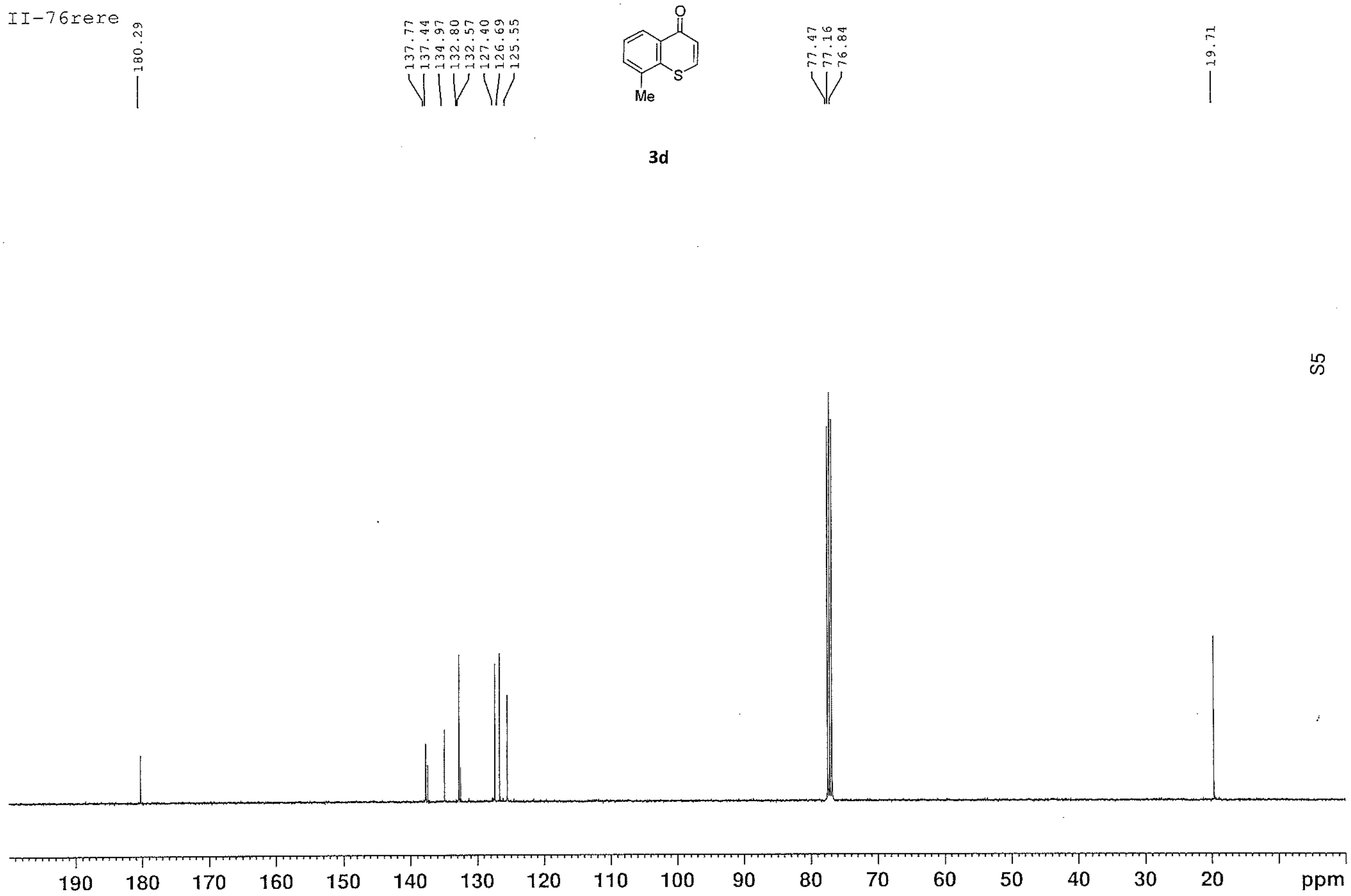
II-76rere
180.29
137.77
137.44
134.97
132.80
132.57
127.40
126.69
125.55
O
S
Me
3d
77.47
77.16
76.84
19.71
190 180 170 160 150 140 130 120 110 100 90 80 70 60 50 40 30 20 ppm

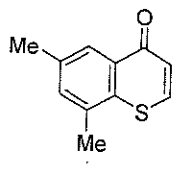

3f

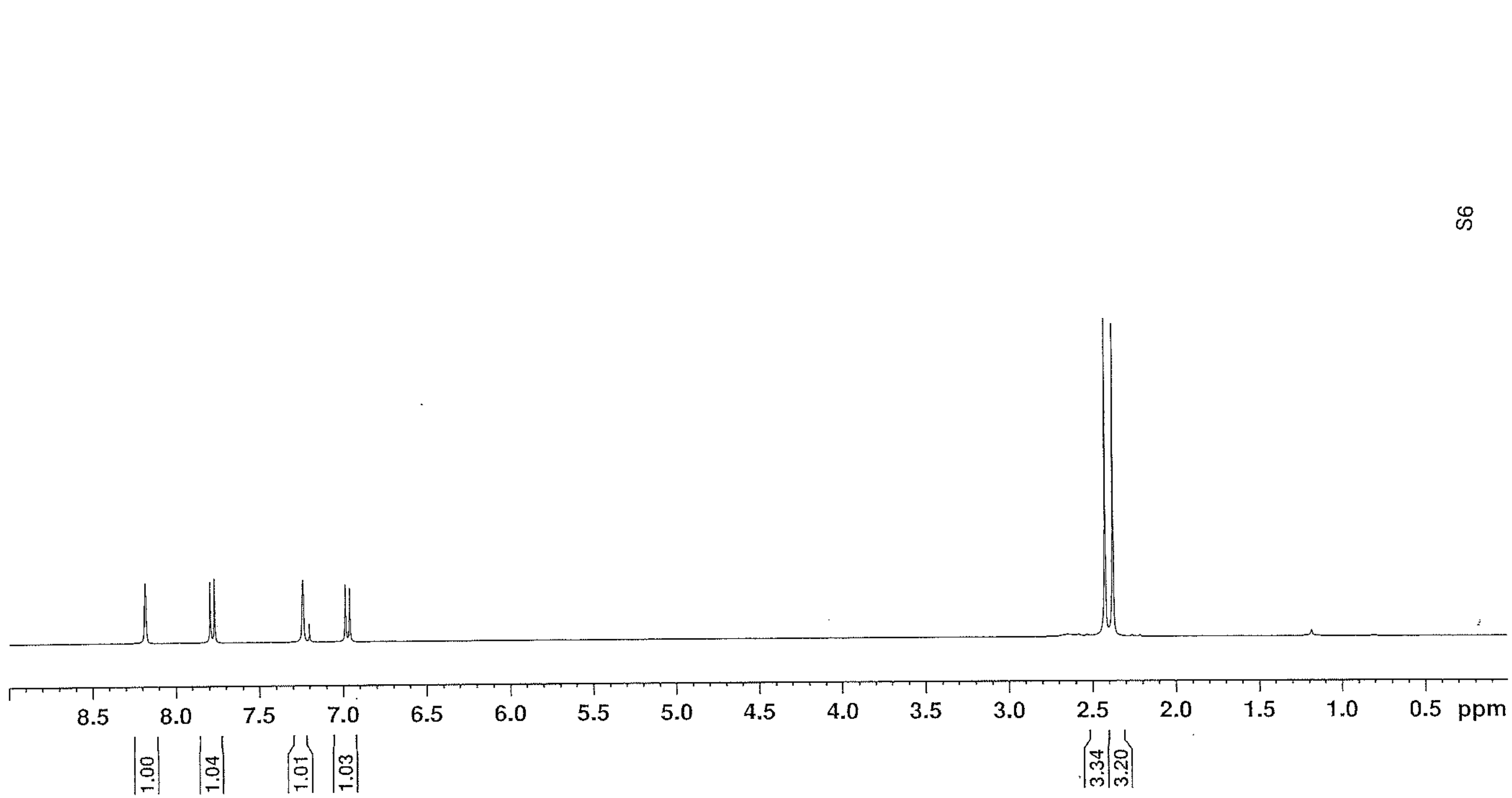
II-98rere
8.183
7.794
7.768
7.240
7.201
6.986
6.960
2.423
2.376
8.5 8.0 7.5 7.0 6.5 6.0 5.5 5.0 4.5 4.0 3.5 3.0 2.5 2.0 1.5 1.0 0.5 ppm
1.00
1.04
1.01
1.03
3.34
3.20

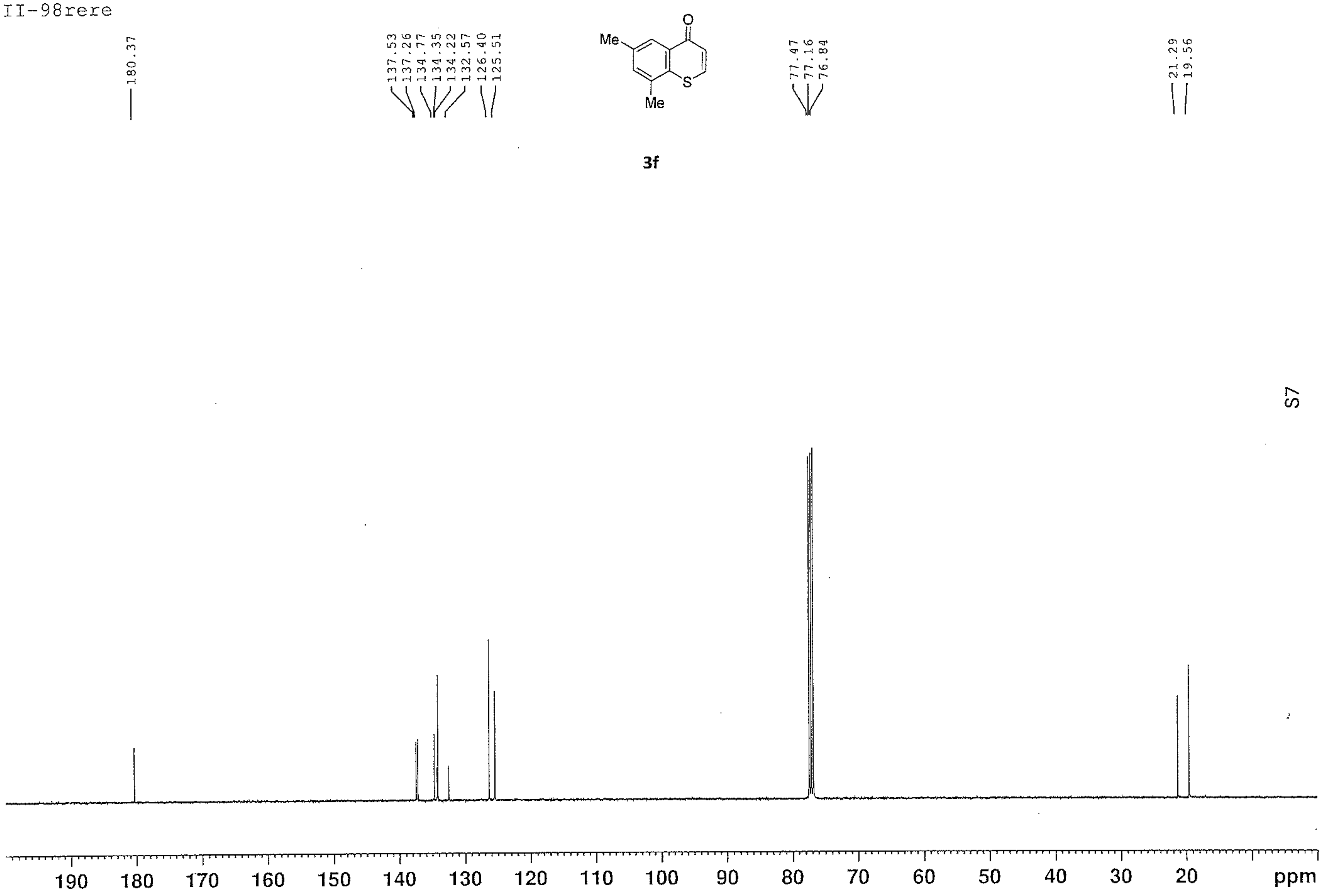
II-98rere
180.37
137.53
137.26
134.77
134.35
134.22
132.57
126.40
125.51
77.47
77.16
76.84
21.29
19.56
Me
Me
O
S
3f
190
180
170
160
150
140
130
120
110
100
90
80
70
60
50
40
30
20
ppm

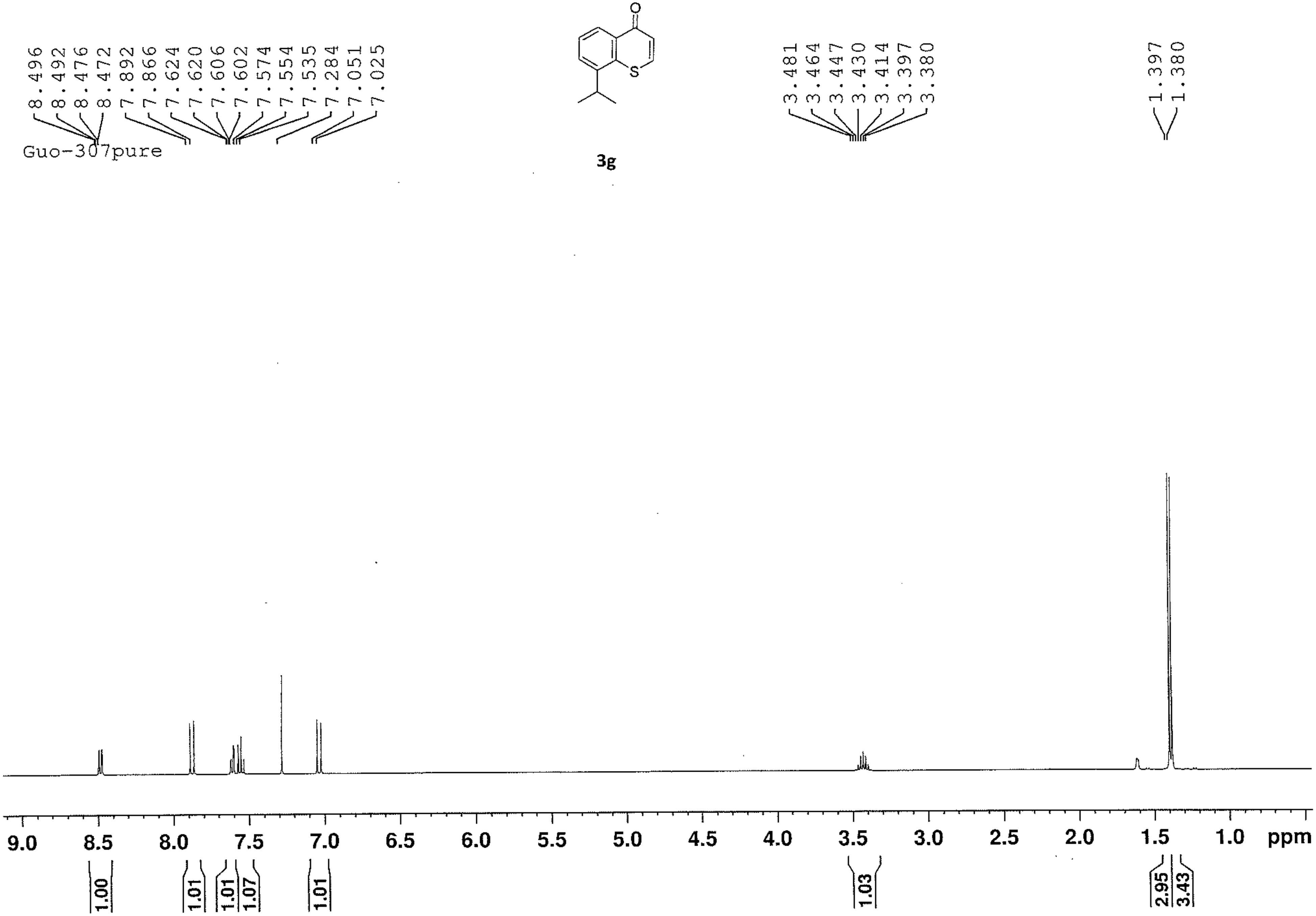
8.496
8.492
8.476
8.472
7.892
7.866
7.624
7.620
7.606
7.602
7.574
7.554
7.535
7.284
7.051
7.025
Guo-307pure
3g
3.481
3.464
3.447
3.430
3.414
3.397
3.380
1.397
1.380
9.0 8.5 8.0 7.5 7.0 6.5 6.0 5.5 5.0 4.5 4.0 3.5 3.0 2.5 2.0 1.5 1.0 ppm
1.00
1.01
1.01
1.07
1.01
1.03
2.95
3.43

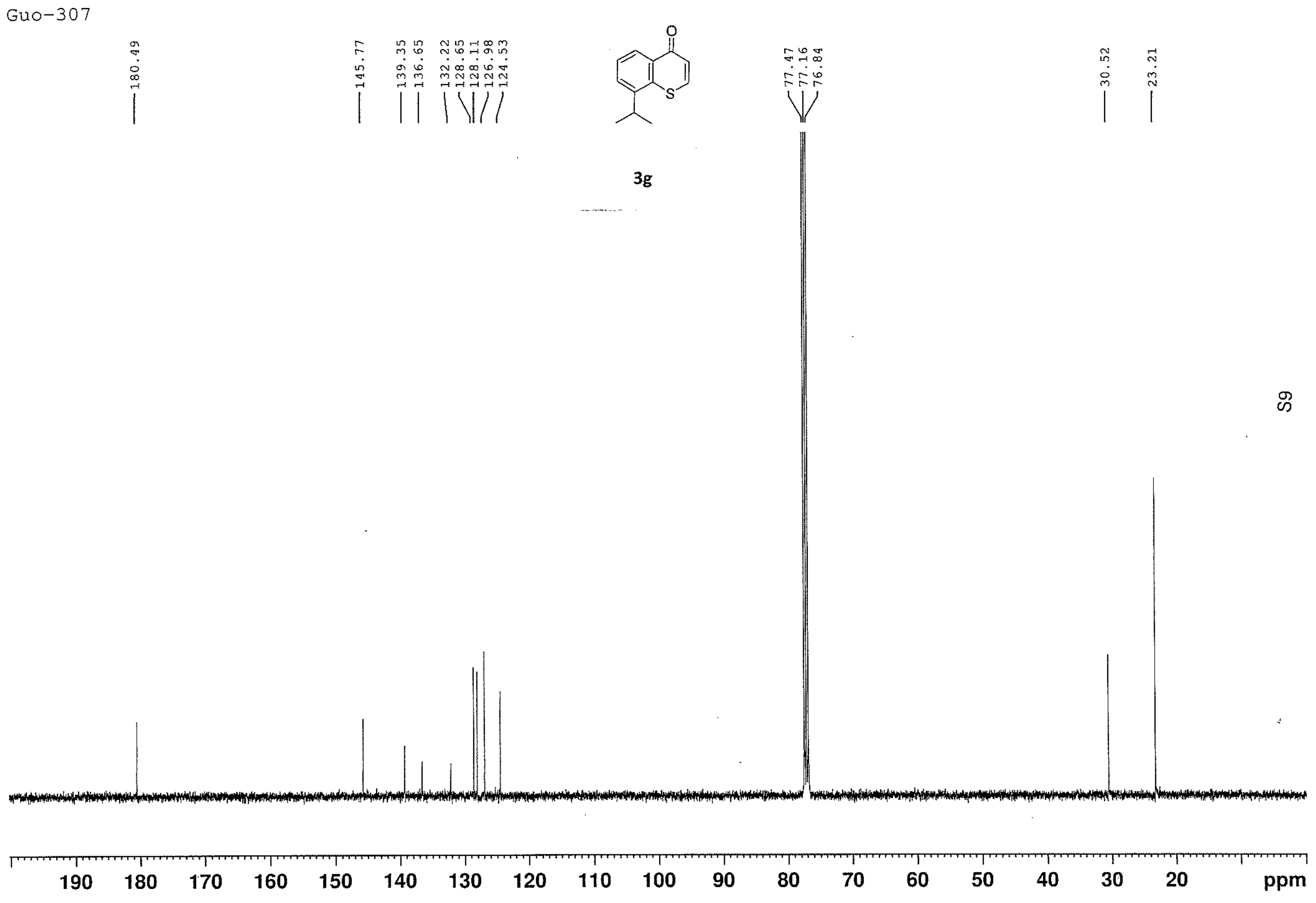
Guo-307
180.49
145.77
139.35
136.65
132.22
128.65
128.11
126.98
124.53
O
S
3g
77.47
77.16
76.84
30.52
23.21
190 180 170 160 150 140 130 120 110 100 90 80 70 60 50 40 30 20 ppm

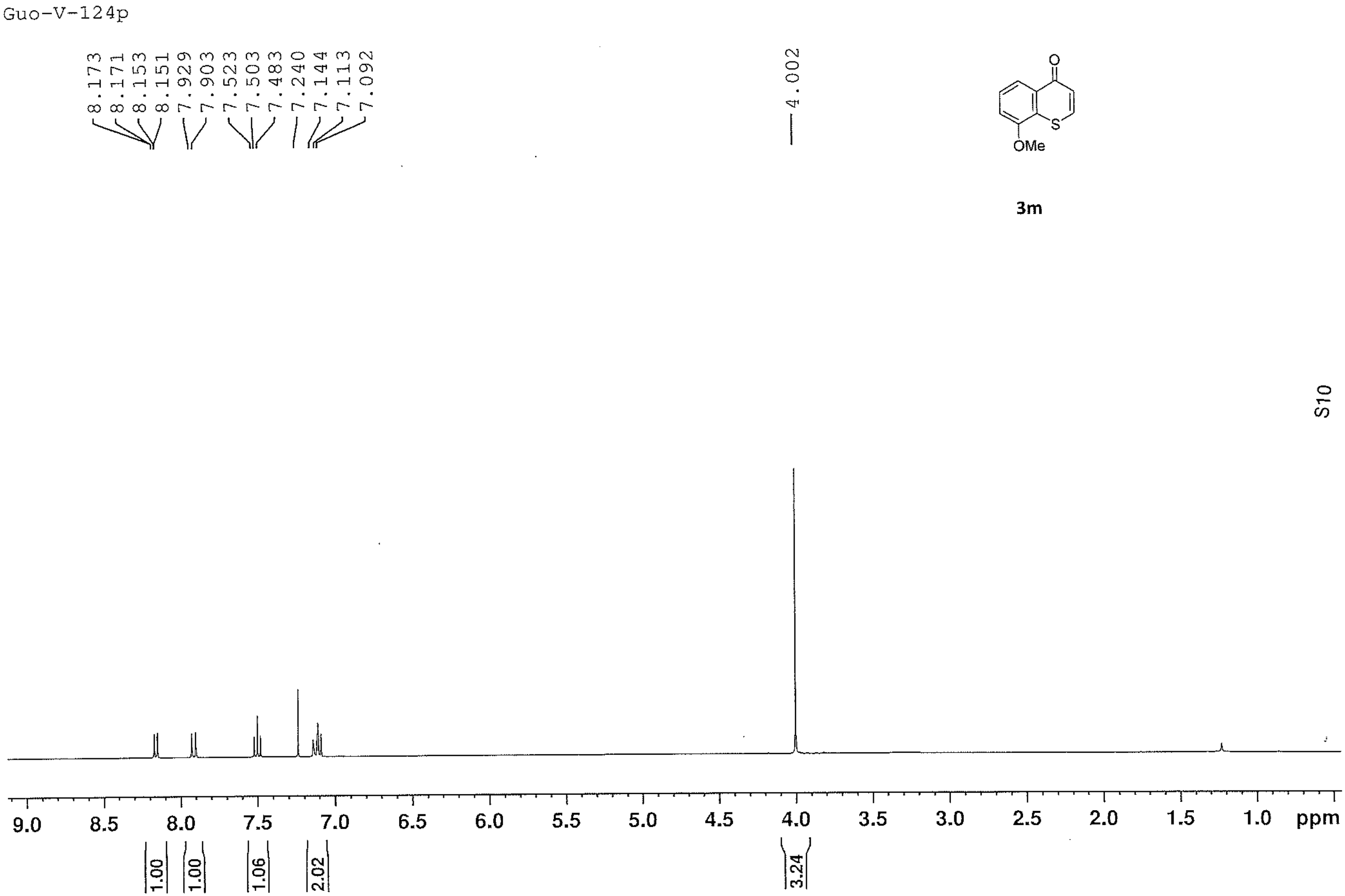
Guo-V-124p
8.173
8.171
8.153
8.151
7.929
7.903
7.523
7.503
7.483
7.240
7.144
7.113
7.092
4.002
O
S
OMe
3m
9.0
8.5
8.0
7.5
7.0
6.5
6.0
5.5
5.0
4.5
4.0
3.5
3.0
2.5
2.0
1.5
1.0
ppm
1.00
1.00
1.06
2.02
3.24

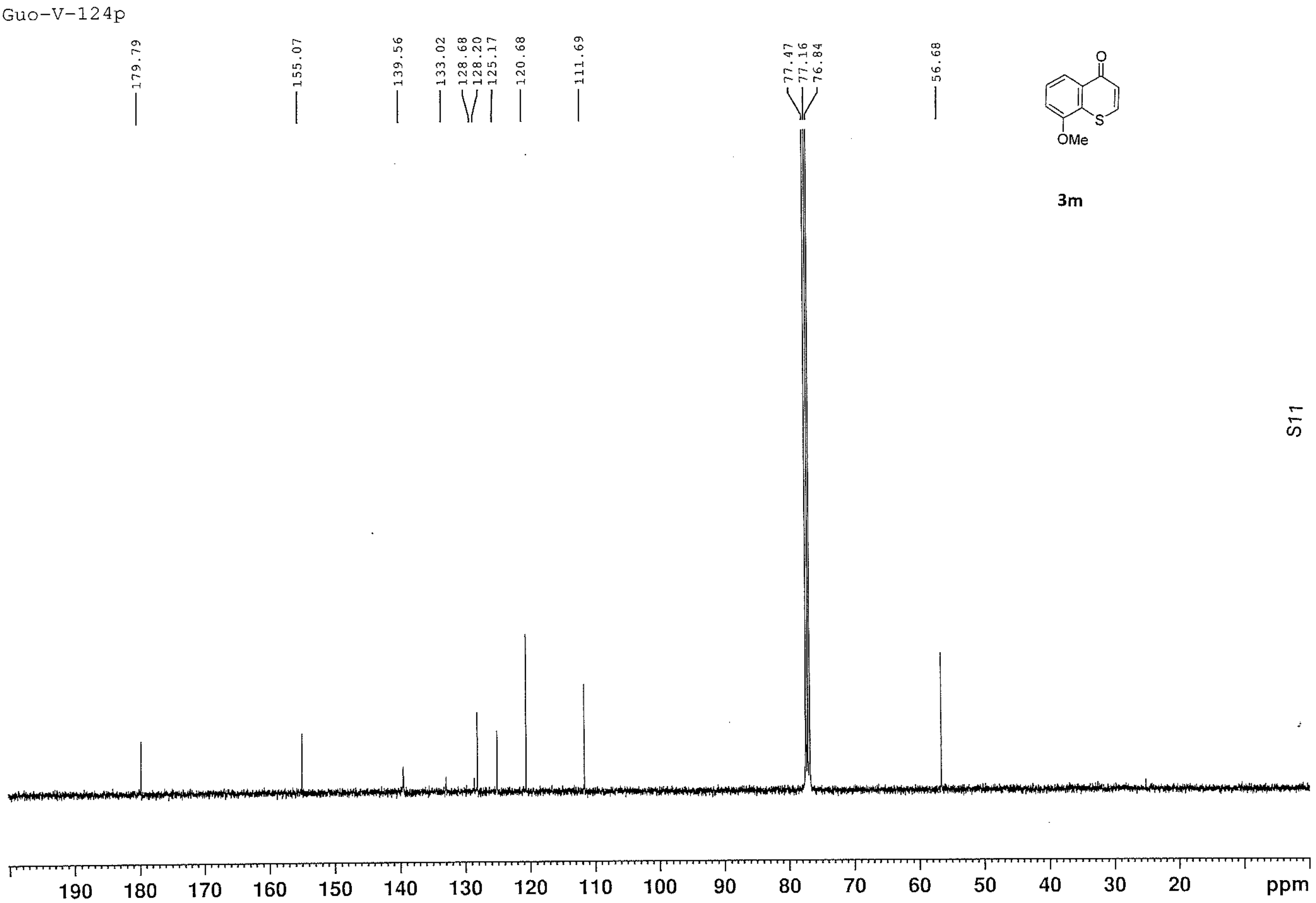

Guo-V-124p
179.79
155.07
139.56
133.02
128.68
128.20
125.17
120.68
111.69
77.47
77.16
76.84
56.68
190
180
170
160
150
140
130
120
110
100
90
80
70
60
50
40
30
20
ppm
O
S
OMe

3m

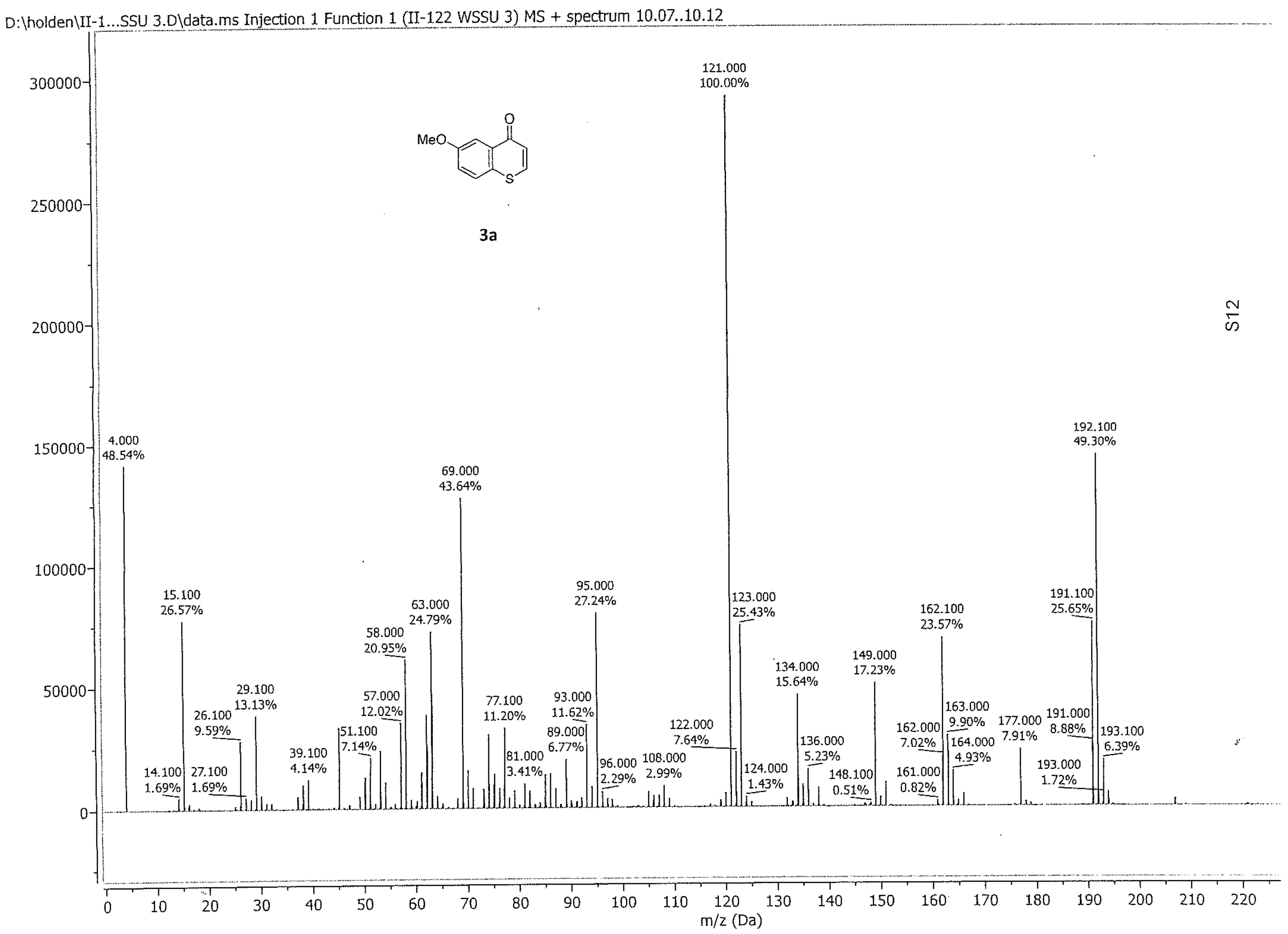
D:\holden\II-1...SSU 3.D\data.ms Injection 1 Function 1 (II-122 WSSU 3) MS + spectrum 10.07..10.12
MeO
O
S
3a
300000
250000
200000
150000
100000
50000
0
4.000 48.54%
14.100 1.69%
15.100 26.57%
26.100 9.59%
27.100 1.69%
29.100 13.13%
39.100 4.14%
51.100 7.14%
57.000 12.02%
58.000 20.95%
63.000 24.79%
69.000 43.64%
77.100 11.20%
81.000 3.41%
89.000 6.77%
93.000 11.62%
95.000 27.24%
96.000 2.29%
108.000 2.99%
121.000 100.00%
122.000 7.64%
123.000 25.43%
124.000 1.43%
134.000 15.64%
136.000 5.23%
148.100 0.51%
149.000 17.23%
161.000 0.82%
162.000 7.02%
162.100 23.57%
163.000 9.90%
164.000 4.93%
177.000 7.91%
191.000 8.88%
191.100 25.65%
192.100 49.30%
193.000 1.72%
193.100 6.39%
0 10 20 30 40 50 60 70 80 90 100 110 120 130 140 150 160 170 180 190 200 210 220
m/z (Da)

:\holden\II-76 WSSU 2.D\data.ms Injection 1 Function 1 (II-76 wssu 2) TIC

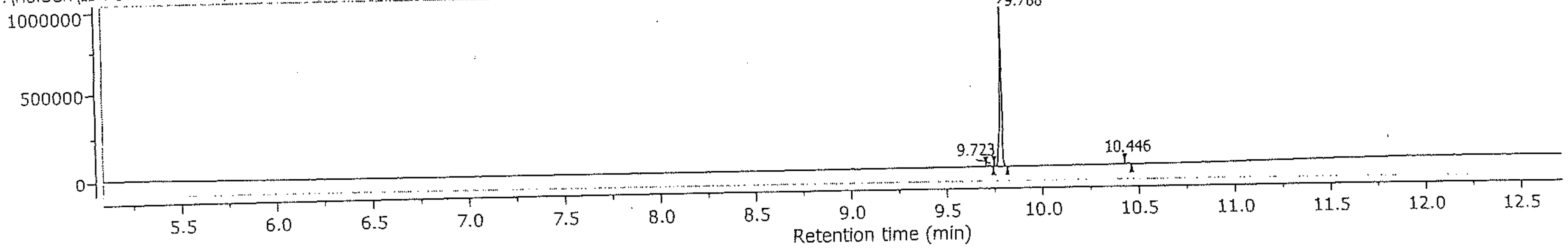

:\holden\II-76 WSSU 2.D\data.ms Injection 1 Function 1 (II-76 wssu 2) MS + spectrum 9.74..9.80

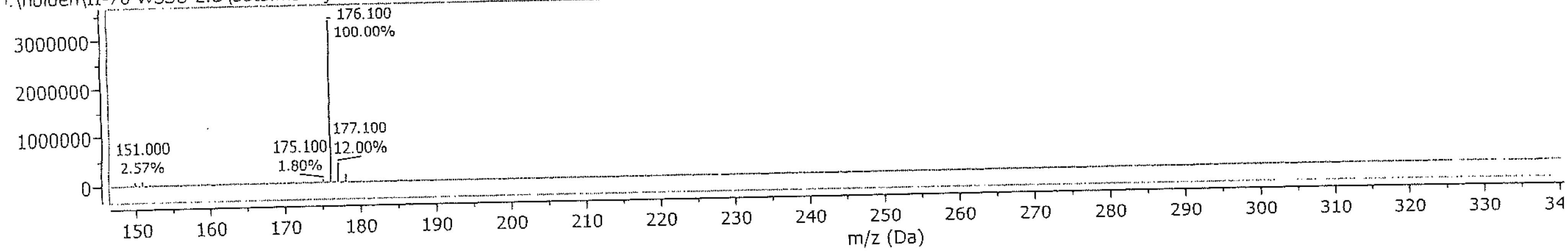

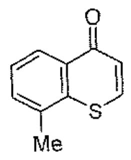

3d

D:\holden\II-98 WSSU 2.D\data.ms Injection 1 Function 1 (II-98 wssu 2) TIC

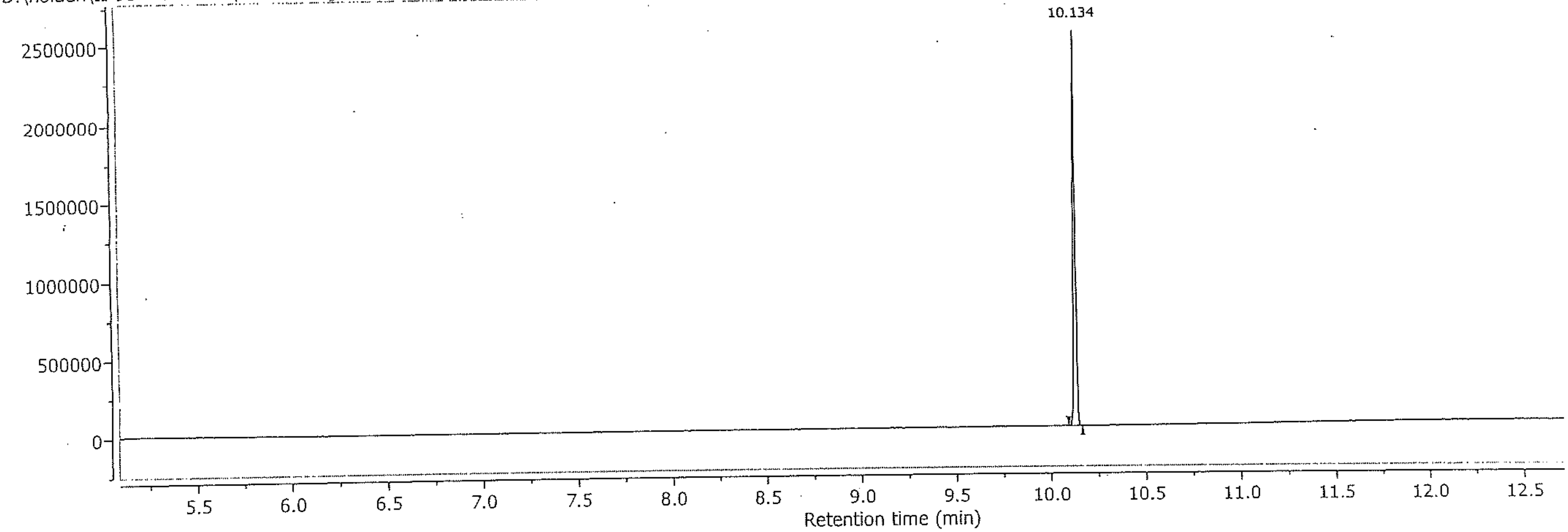

D:\holden\II-98 WSSU 2.D\data.ms Injection 1 Function 1 (II-98 wssu 2) MS + spectrum 10.09..10.16

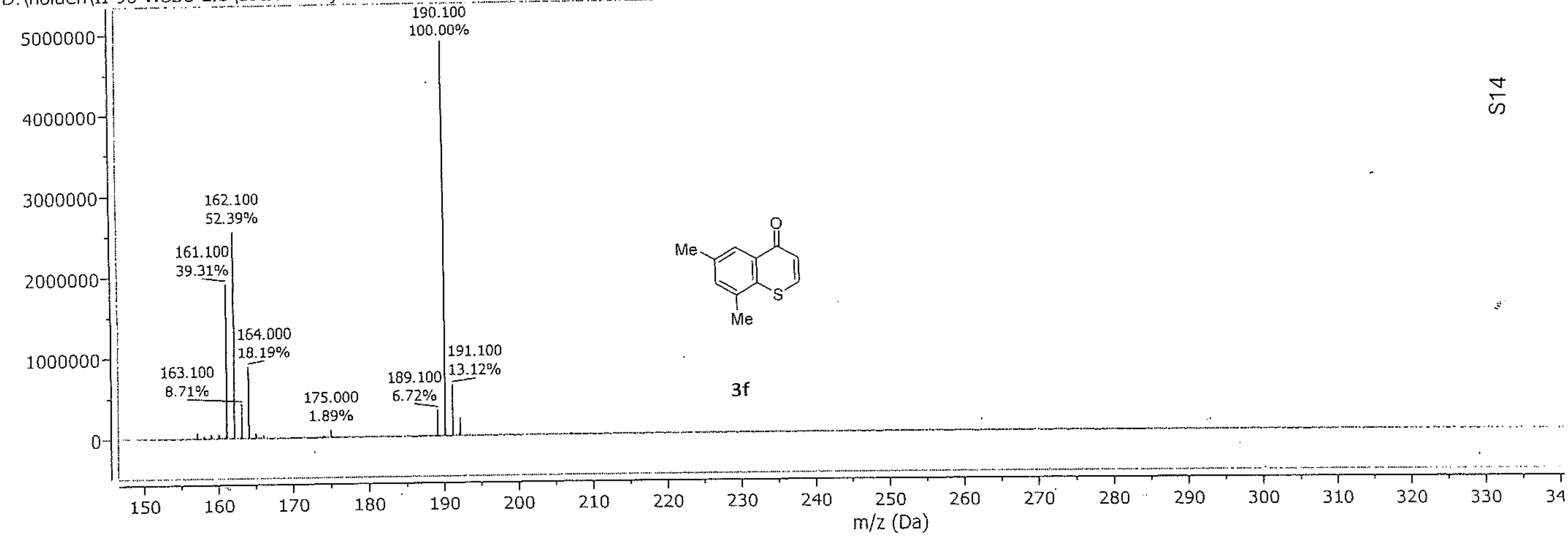

3f

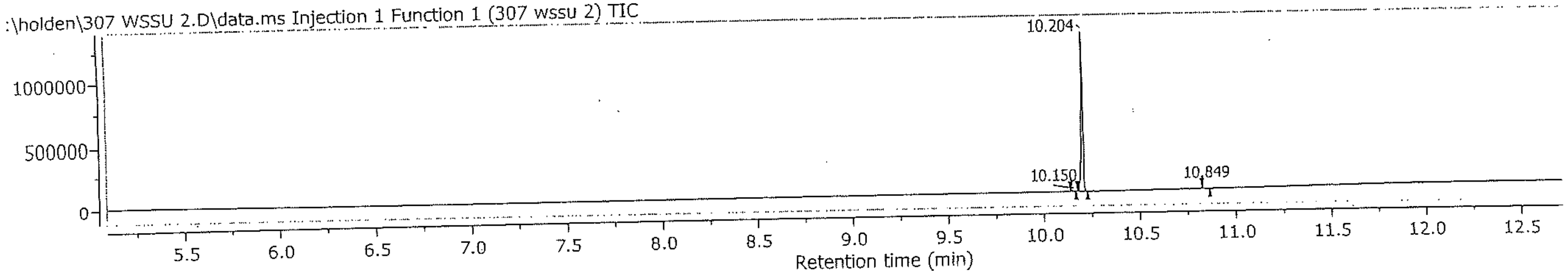
:\holden\307 WSSU 2.D\data.ms Injection 1 Function 1 (307 wssu 2) TIC
10.204
10.150
10.849
1000000
500000
0
5.5
6.0
6.5
7.0
7.5
8.0
8.5
9.0
9.5
10.0
10.5
11.0
11.5
12.0
12.5
Retention time (min)

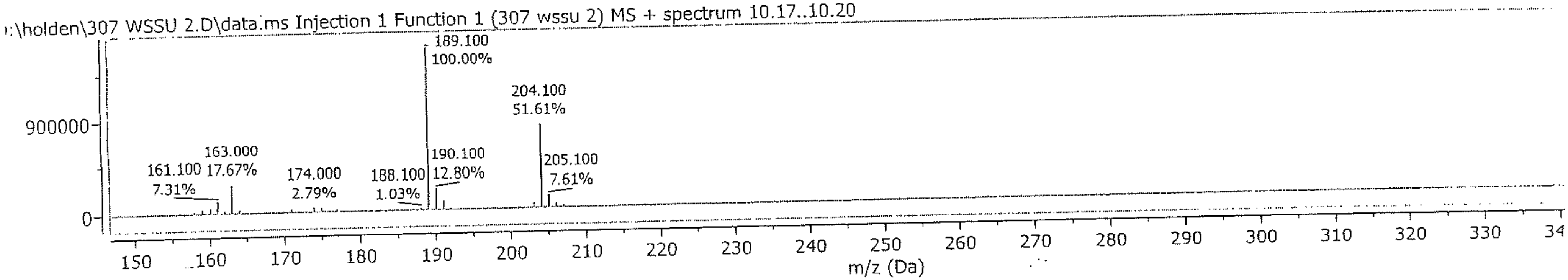
:\holden\307 WSSU 2.D\data.ms Injection 1 Function 1 (307 wssu 2) MS + spectrum 10.17..10.20
189.100
100.00%
204.100
51.61%
163.000
161.100 17.67%
7.31%
174.000
2.79%
188.100
1.03%
190.100
12.80%
205.100
7.61%
900000
0
150
160
170
180
190
200
210
220
230
240
250
260
270
280
290
300
310
320
330
34
m/z (Da)

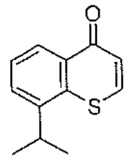
O
S

3g

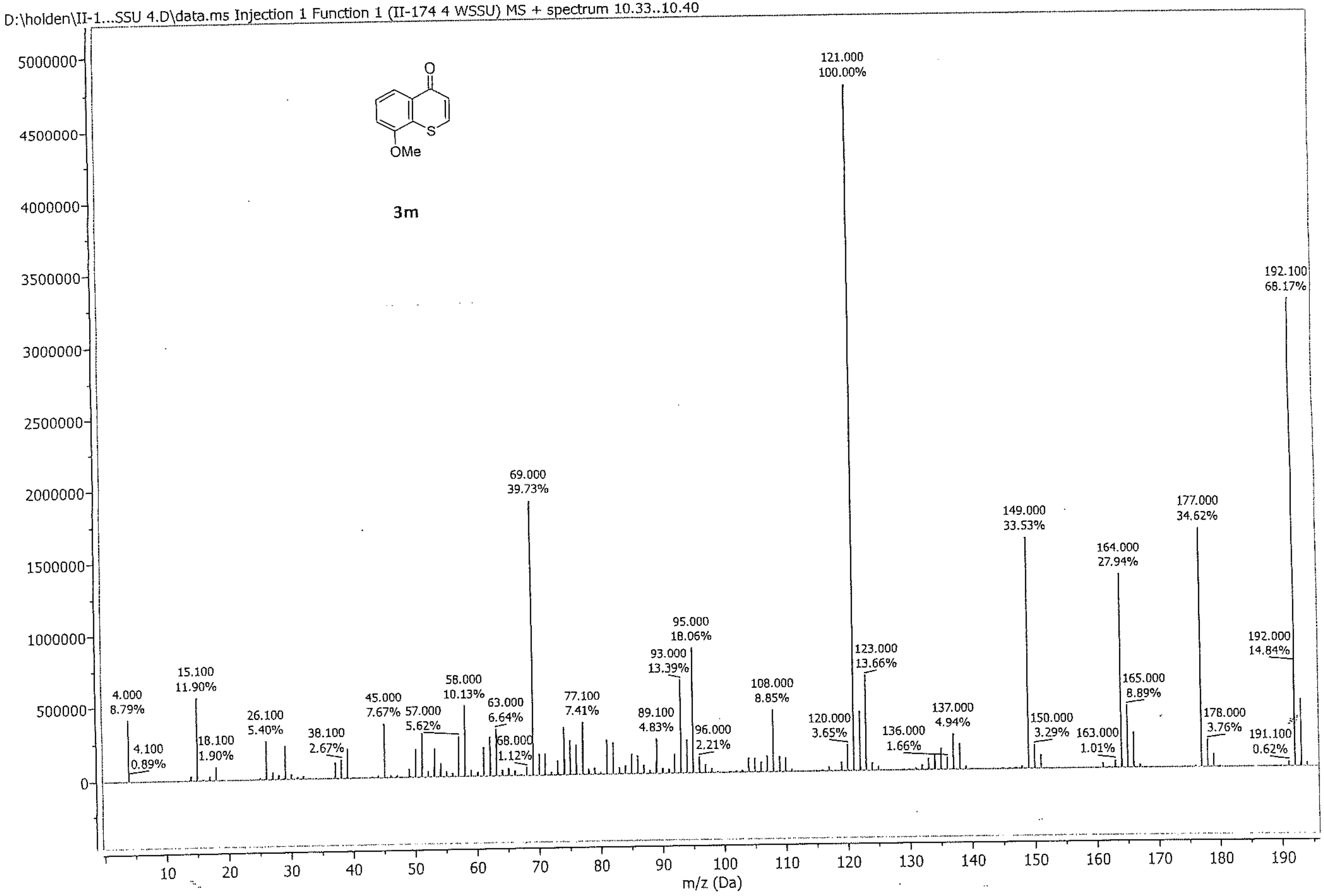
D:\holden\II-1...SSU 4.D\data.ms Injection 1 Function 1 (II-174 4 WSSU) MS + spectrum 10.33..10.40
OMe
S
O
3m
121.000
100.00%
192.100
68.17%
69.000
39.73%
177.000
34.62%
149.000
33.53%
164.000
27.94%
95.000
18.06%
192.000
14.84%
123.000
13.66%
93.000
13.39%
15.100
11.90%
58.000
10.13%
165.000
8.89%
108.000
8.85%
4.000
8.79%
45.000
7.67%
77.100
7.41%
63.000
6.64%
57.000
5.62%
26.100
5.40%
137.000
4.94%
89.100
4.83%
178.000
3.76%
120.000
3.65%
150.000
3.29%
38.100
2.67%
96.000
2.21%
18.100
1.90%
136.000
1.66%
68.000
1.12%
163.000
1.01%
4.100
0.89%
191.100
0.62%
m/z (Da)

162rerep-H

8.529
7.864
7.838
7.619
7.616
7.607
7.604
7.564
7.555
7.552
7.543
7.535
7.531
7.523
7.240
7.084
7.058

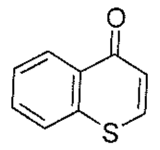

3b

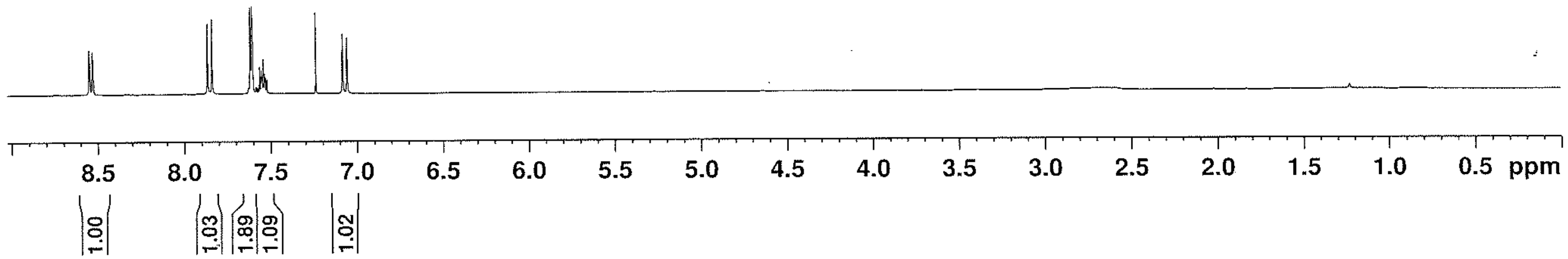

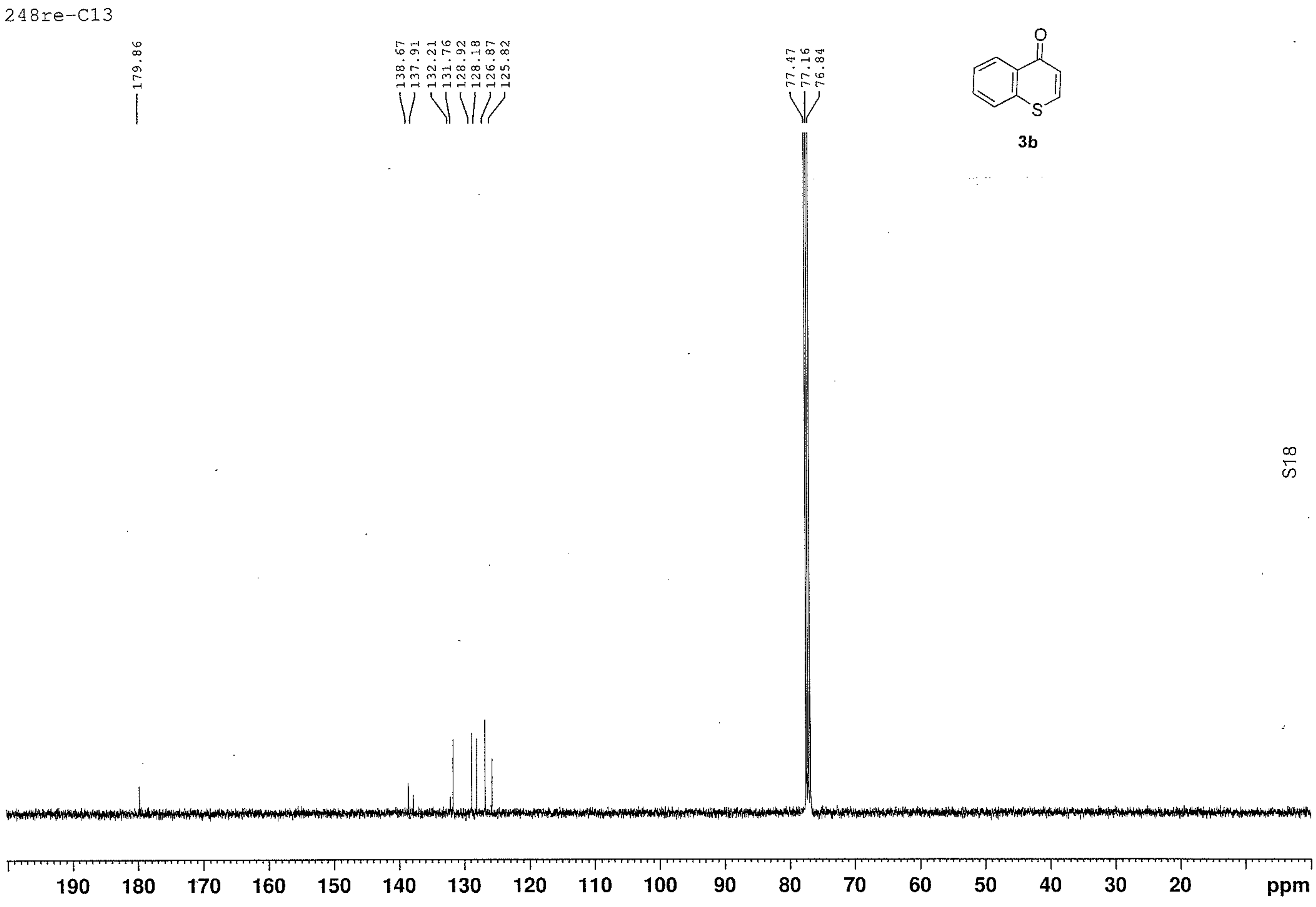
248re-C13
179.86
138.67
137.91
132.21
131.76
128.92
128.18
126.87
125.82
77.47
77.16
76.84
O
S
3b
190 180 170 160 150 140 130 120 110 100 90 80 70 60 50 40 30 20 ppm

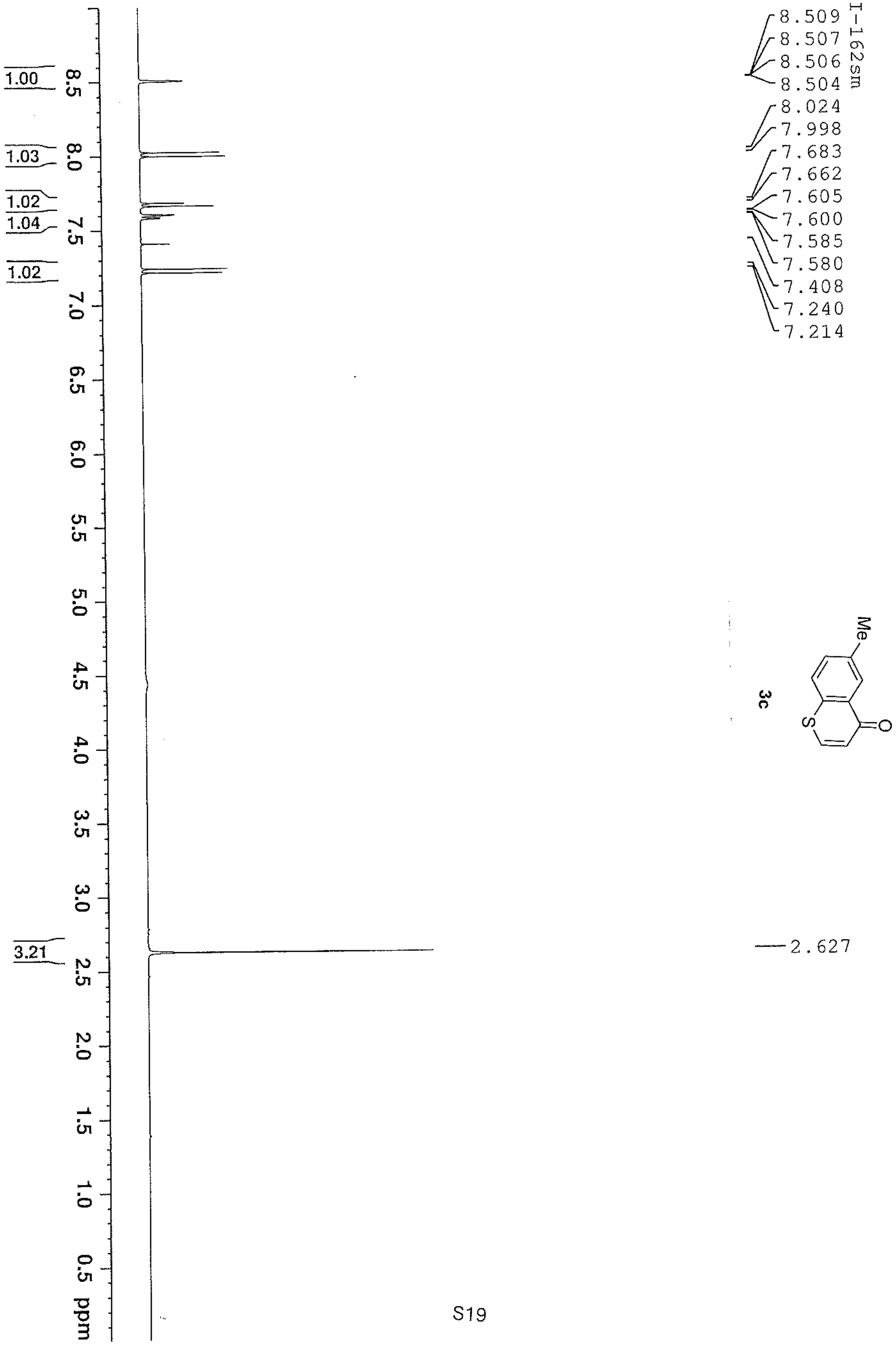
I-162sm
8.509
8.507
8.506
8.504
8.024
7.998
7.683
7.662
7.605
7.600
7.585
7.580
7.408
7.240
7.214
2.627
1.00
1.03
1.02
1.04
1.02
3.21
8.5
8.0
7.5
7.0
6.5
6.0
5.5
5.0
4.5
4.0
3.5
3.0
2.5
2.0
1.5
1.0
0.5 ppm
Me
S
O
3c

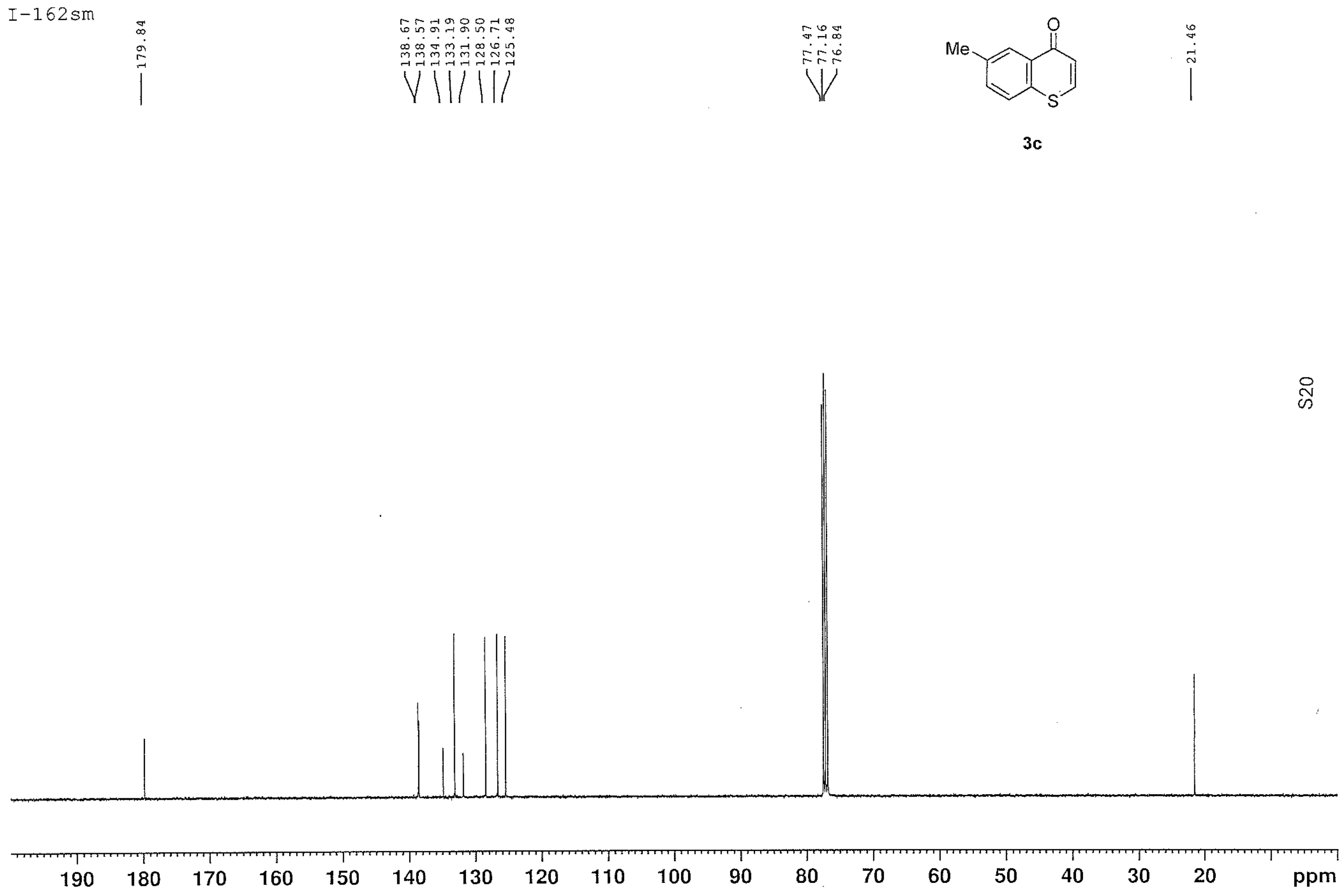
I-162sm
179.84
138.67
138.57
134.91
133.19
131.90
128.50
126.71
125.48
77.47
77.16
76.84
Me
O
S
3c
21.46
190 180 170 160 150 140 130 120 110 100 90 80 70 60 50 40 30 20 ppm

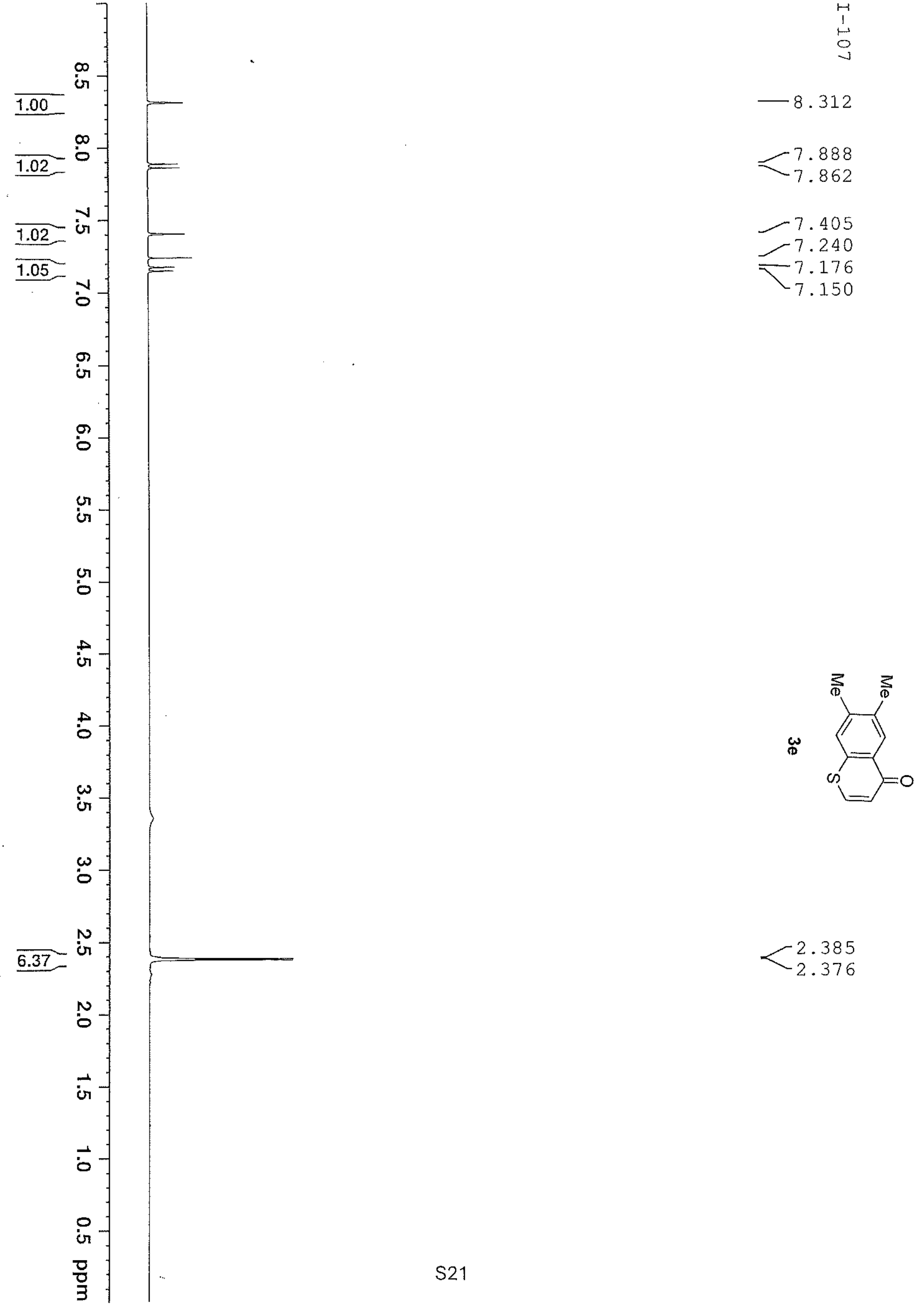
I-107
8.312
7.888
7.862
7.405
7.240
7.176
7.150
2.385
2.376
1.00
1.02
1.02
1.05
6.37
3e
Me
Me
S
O
8.5
8.0
7.5
7.0
6.5
6.0
5.5
5.0
4.5
4.0
3.5
3.0
2.5
2.0
1.5
1.0
0.5 ppm

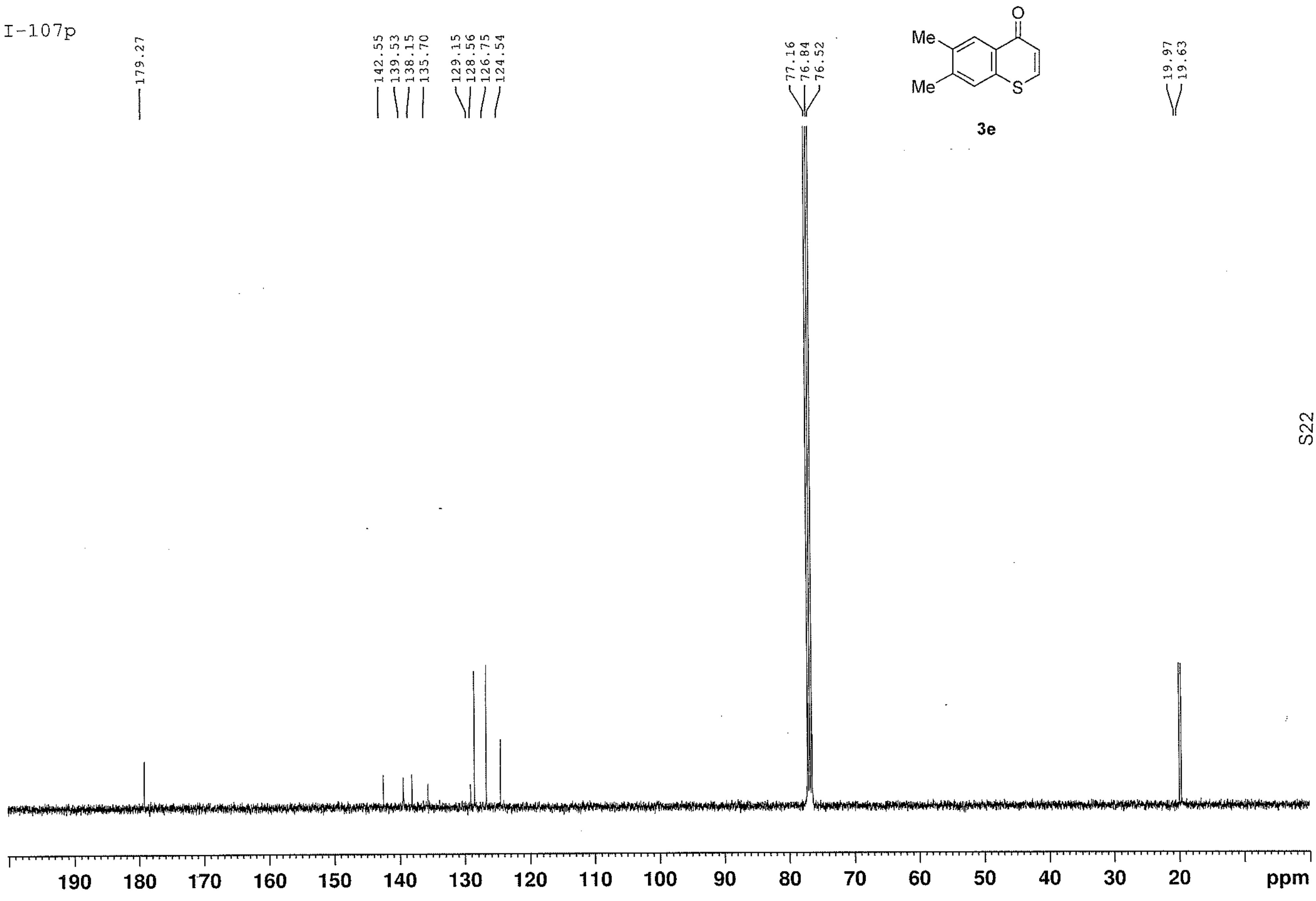
I-107p
179.27
142.55
139.53
138.15
135.70
129.15
128.56
126.75
124.54
77.16
76.84
76.52
19.97
19.63
Me
Me
O
S
3e
190 180 170 160 150 140 130 120 110 100 90 80 70 60 50 40 30 20 ppm

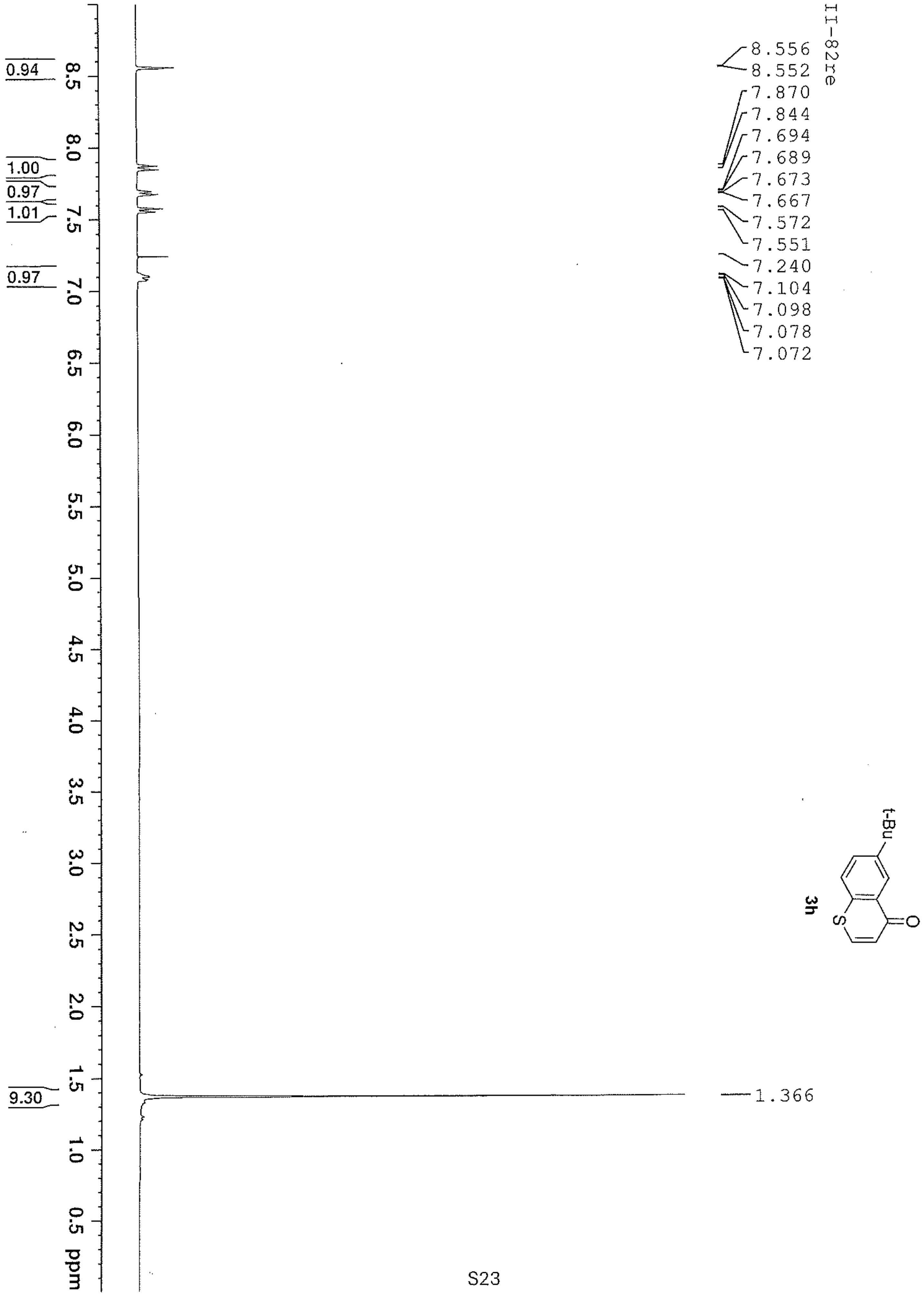
II-82re
8.556
8.552
7.870
7.844
7.694
7.689
7.673
7.667
7.572
7.551
7.240
7.104
7.098
7.078
7.072
1.366
0.94
1.00
0.97
1.01
0.97
9.30
8.5
8.0
7.5
7.0
6.5
6.0
5.5
5.0
4.5
4.0
3.5
3.0
2.5
2.0
1.5
1.0
0.5 ppm
t-Bu
S
O
3h

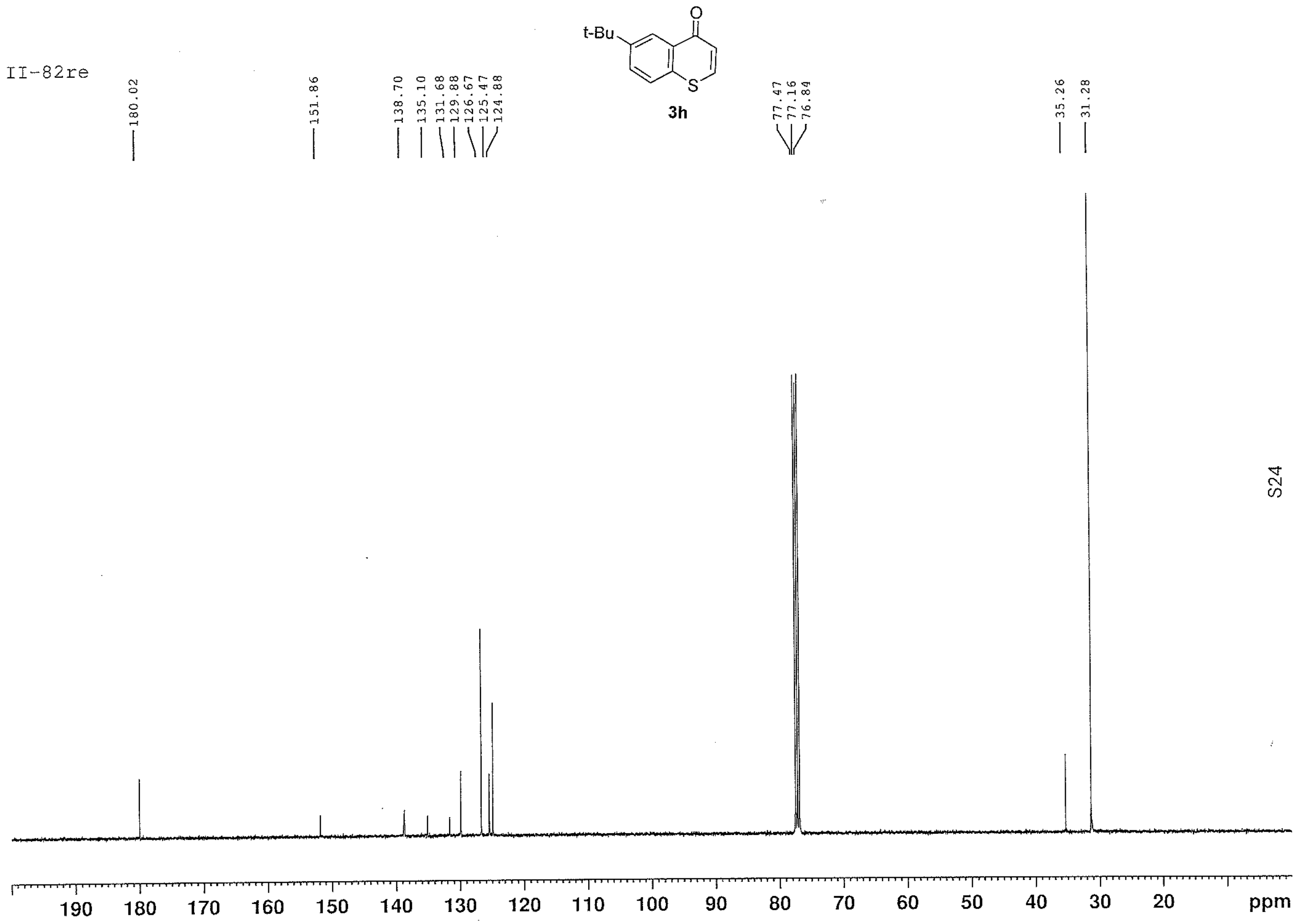
II-82re
180.02
151.86
138.70
135.10
131.68
129.88
126.67
125.47
124.88
t-Bu
O
S
3h
77.47
77.16
76.84
35.26
31.28
190
180
170
160
150
140
130
120
110
100
90
80
70
60
50
40
30
20
ppm

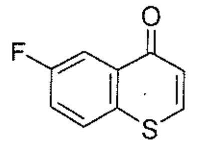

3i

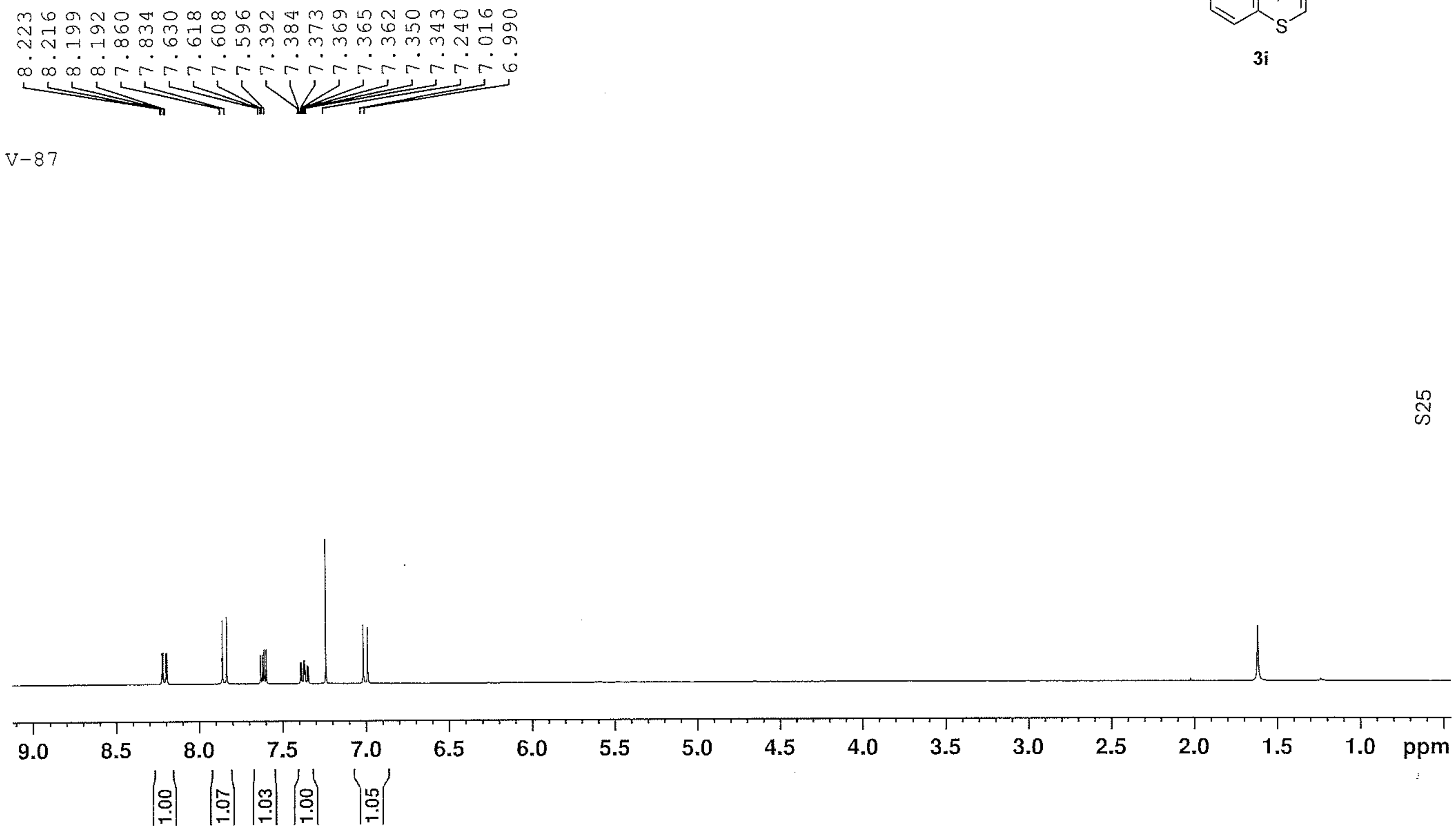

8.223
8.216
8.199
8.192
7.860
7.834
7.630
7.618
7.608
7.596
7.392
7.384
7.373
7.369
7.365
7.362
7.350
7.343
7.240
7.016
6.990
V-87
9.0
8.5
8.0
7.5
7.0
6.5
6.0
5.5
5.0
4.5
4.0
3.5
3.0
2.5
2.0
1.5
1.0
ppm
1.00
1.07
1.03
1.00
1.05

v-87

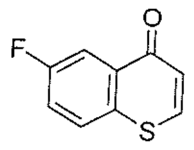

3i

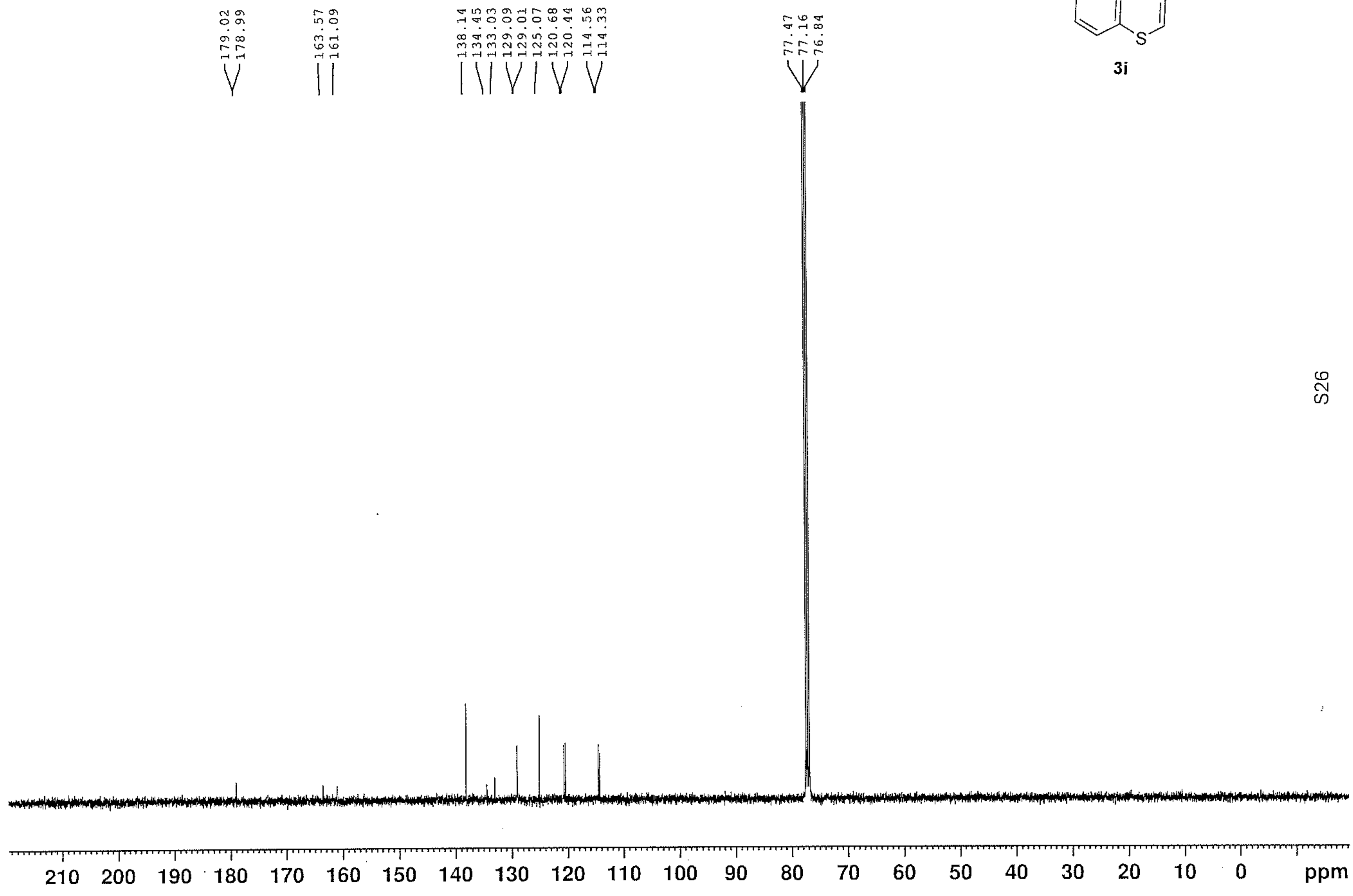

179.02
178.99
163.57
161.09
138.14
134.45
133.03
129.09
129.01
125.07
120.68
120.44
114.56
114.33
77.47
77.16
76.84
210 200 190 180 170 160 150 140 130 120 110 100 90 80 70 60 50 40 30 20 10 0 ppm

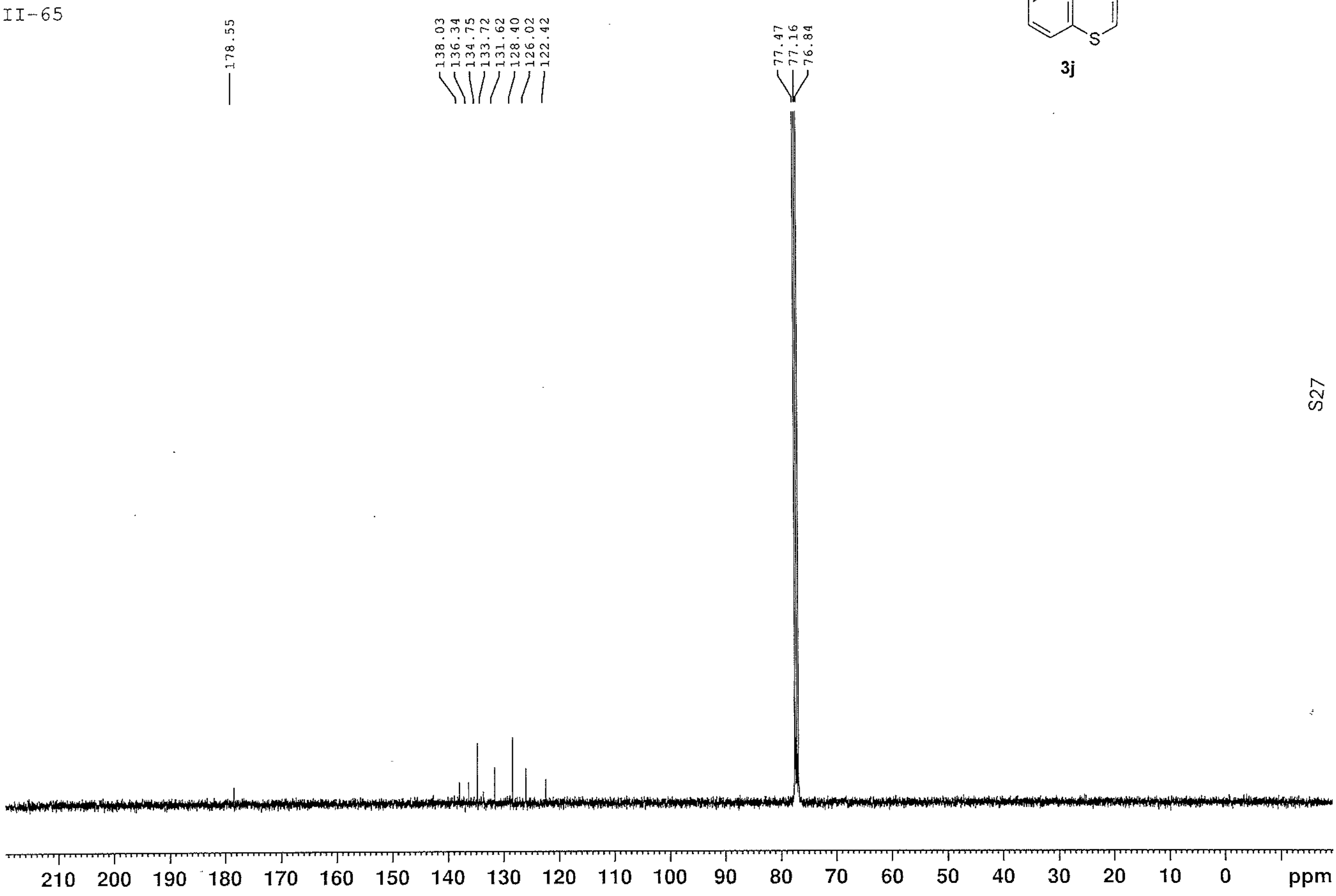
II-65
178.55
138.03
136.34
134.75
133.72
131.62
128.40
126.02
122.42
77.47
77.16
76.84
210 200 190 180 170 160 150 140 130 120 110 100 90 80 70 60 50 40 30 20 10 0 ppm

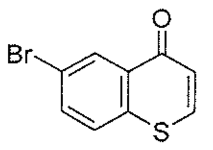
Br
O
S

3j

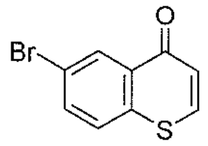

Br
O
S

3j

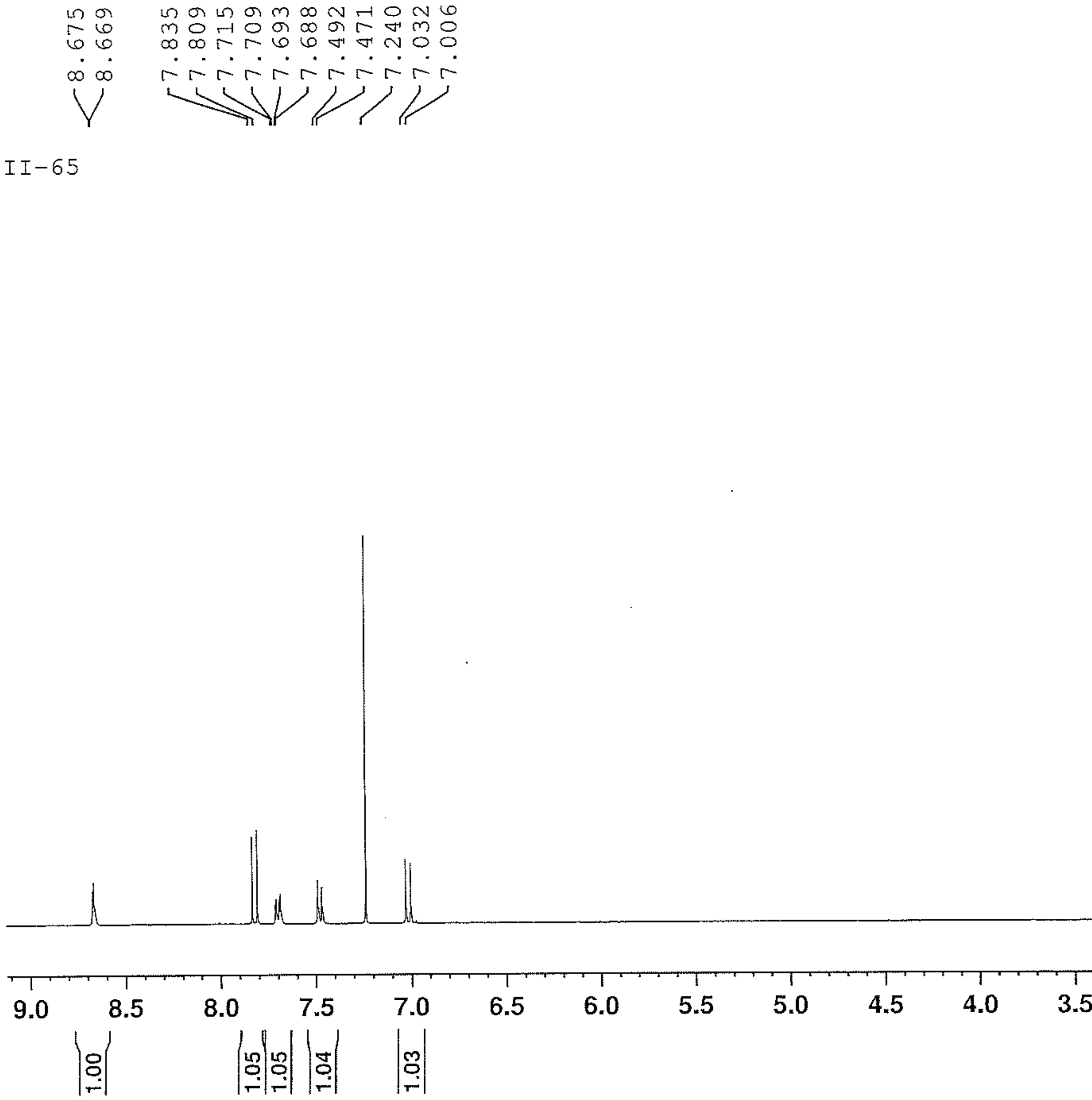

II-65
8.675
8.669
7.835
7.809
7.715
7.709
7.693
7.688
7.492
7.471
7.240
7.032
7.006
9.0
8.5
8.0
7.5
7.0
6.5
6.0
5.5
5.0
4.5
4.0
3.5
3.0
2.5
2.0
1.5
1.0
ppm
1.00
1.05
1.05
1.04
1.03

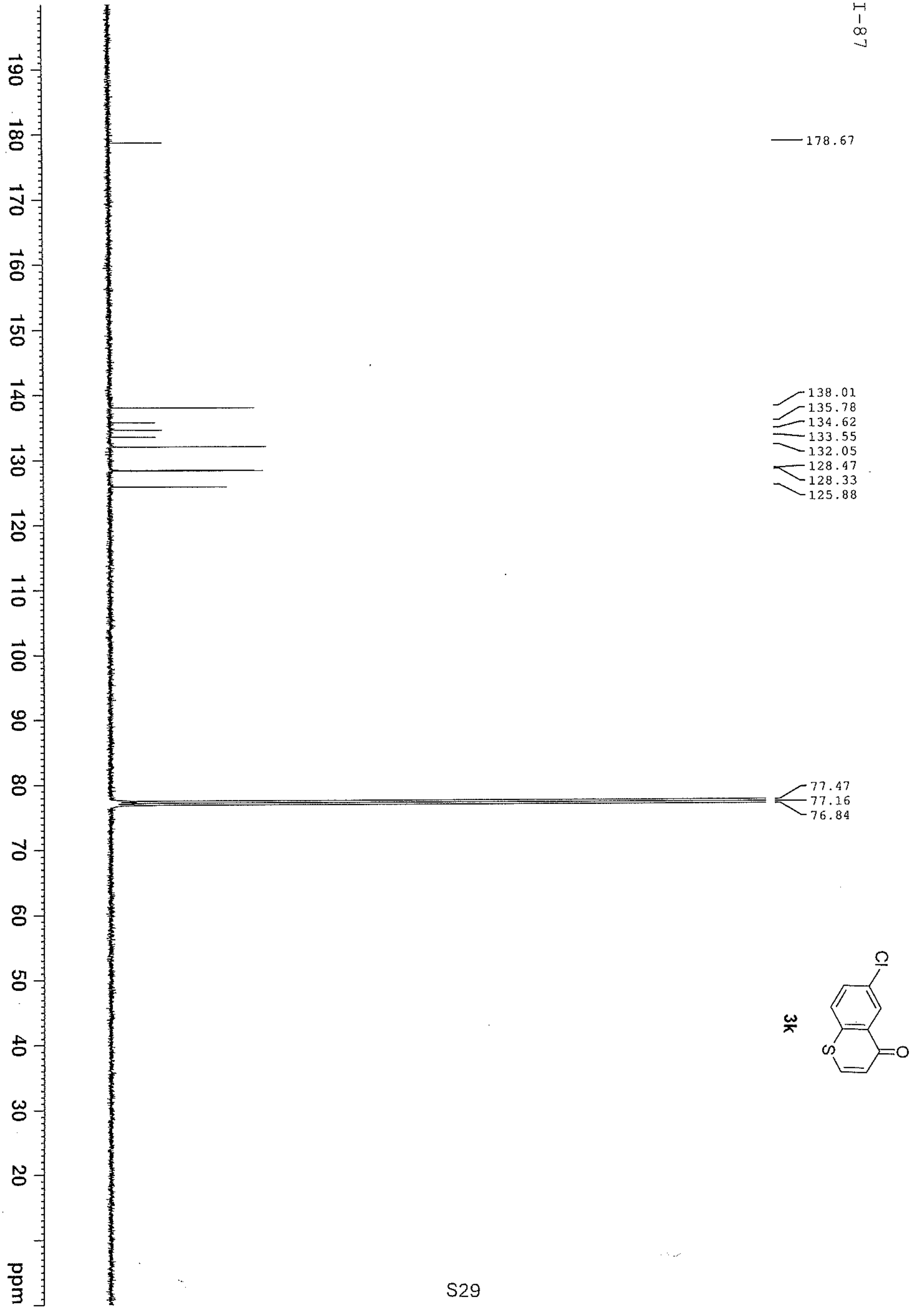
I-87
178.67
138.01
135.78
134.62
133.55
132.05
128.47
128.33
125.88
77.47
77.16
76.84
3k
Cl
S
O
190
180
170
160
150
140
130
120
110
100
90
80
70
60
50
40
30
20
ppm

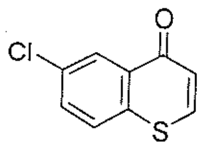

3k

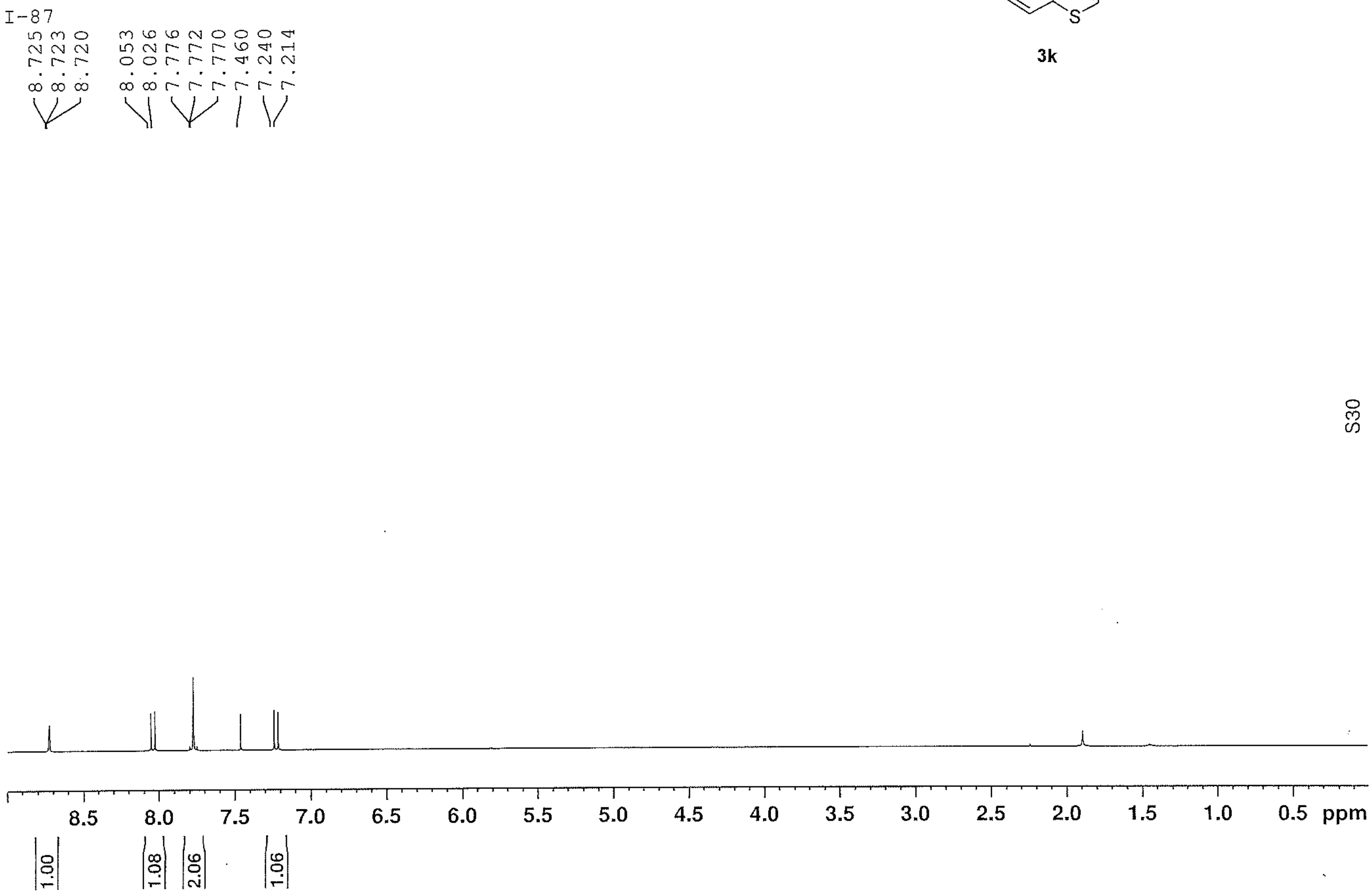
I-87
8.725
8.723
8.720
8.053
8.026
7.776
7.772
7.770
7.460
7.240
7.214
8.5
8.0
7.5
7.0
6.5
6.0
5.5
5.0
4.5
4.0
3.5
3.0
2.5
2.0
1.5
1.0
0.5
ppm
1.00
1.08
2.06
1.06

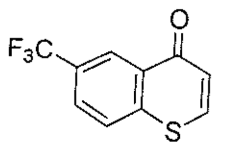
F3C
O
S

3l

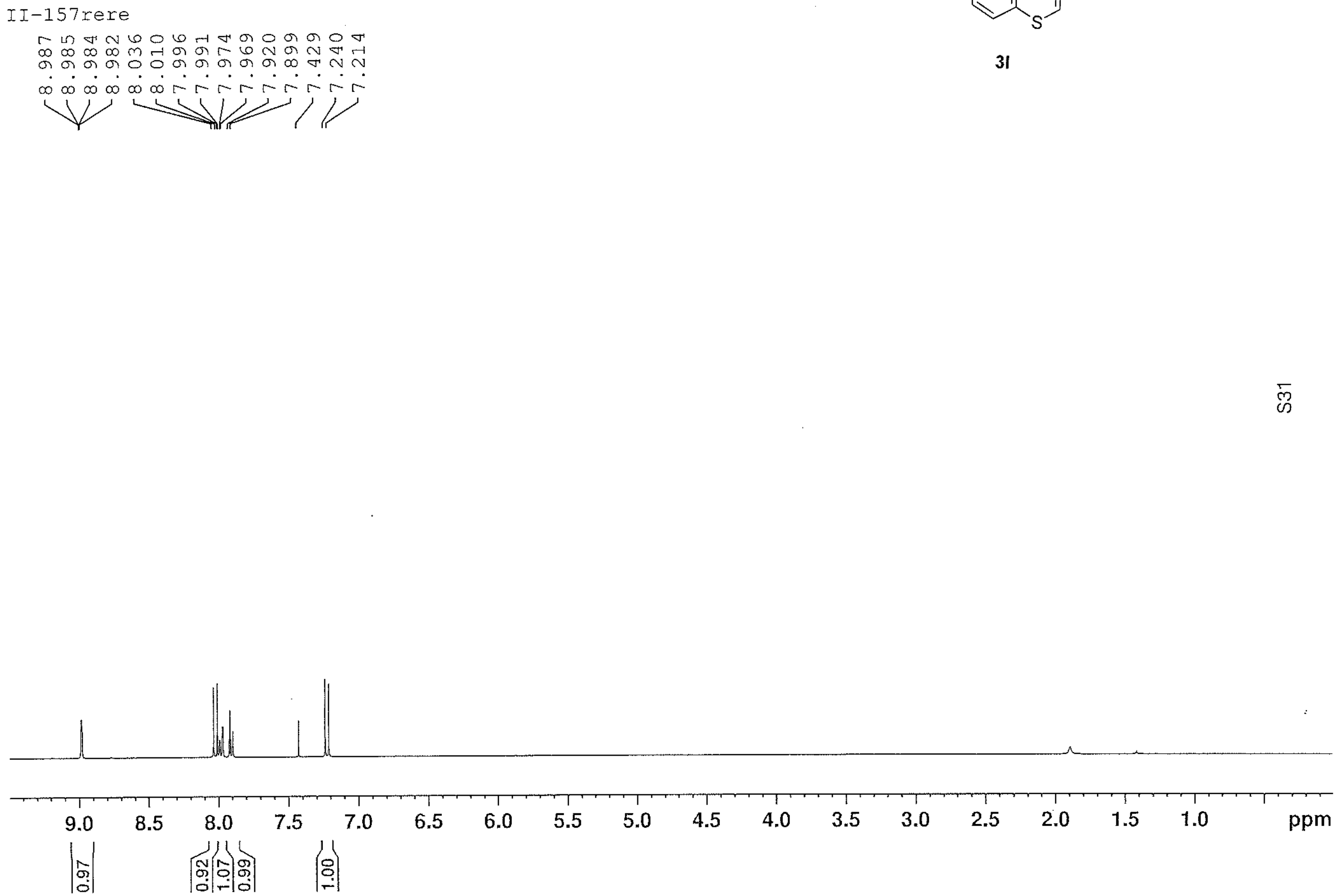
II-157rere
8.987
8.985
8.984
8.982
8.036
8.010
7.996
7.991
7.974
7.969
7.920
7.899
7.429
7.240
7.214
9.0
8.5
8.0
7.5
7.0
6.5
6.0
5.5
5.0
4.5
4.0
3.5
3.0
2.5
2.0
1.5
1.0
ppm
0.97
0.92
1.07
0.99
1.00

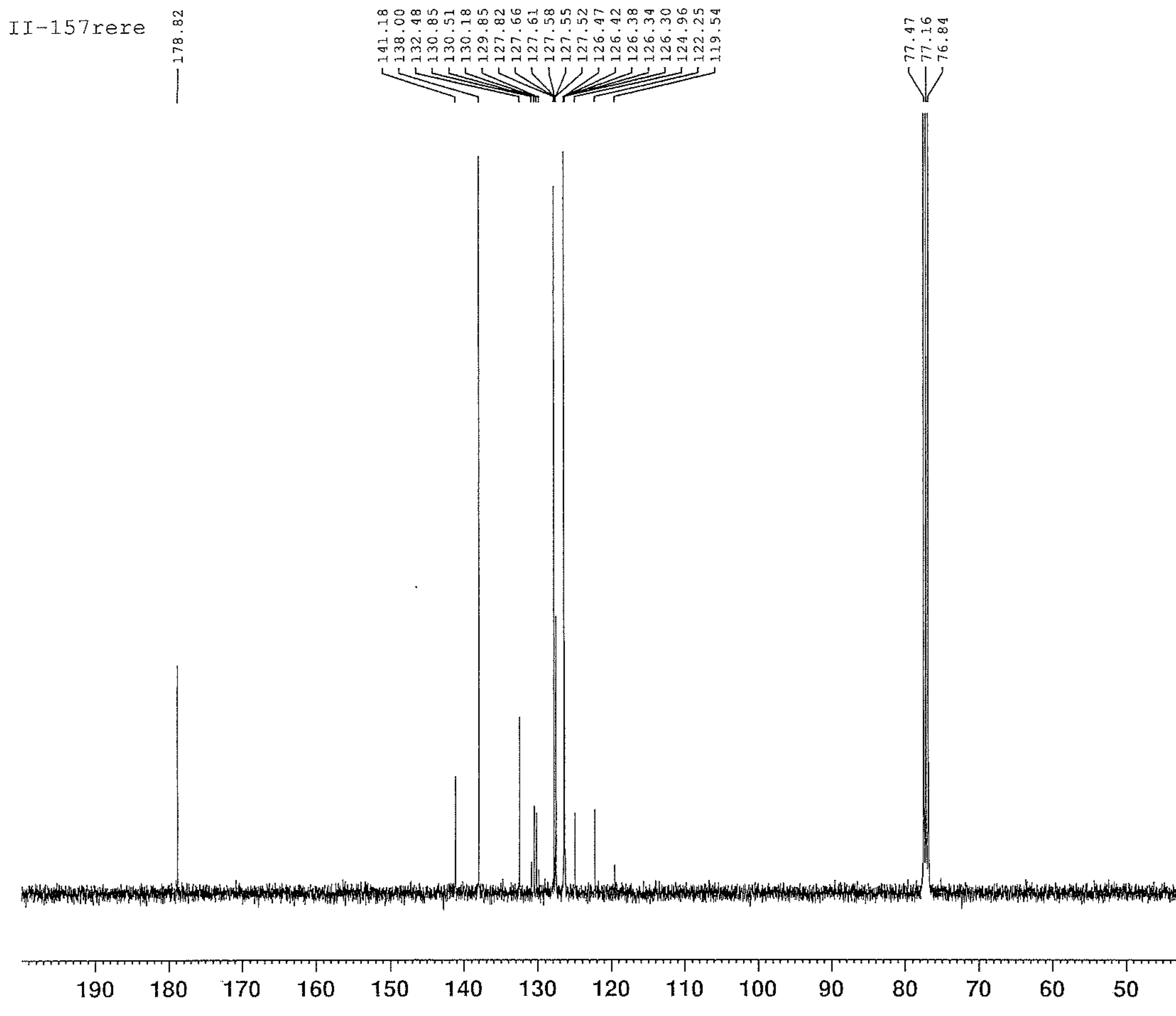
II-157rere
178.82
141.18
138.00
132.48
130.85
130.51
130.18
129.85
127.82
127.66
127.61
127.58
127.55
127.52
126.47
126.42
126.38
126.34
126.30
124.96
122.25
119.54
77.47
77.16
76.84
190 180 170 160 150 140 130 120 110 100 90 80 70 60 50 40 30 20 ppm

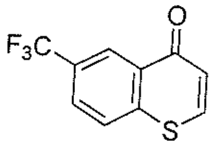
F3C
O
S

3l
